# Inflammatory Cytokines and Risk of Ischemic Stroke: A Mendelian Randomization Study

**DOI:** 10.3389/fphar.2021.779899

**Published:** 2022-01-17

**Authors:** Yalan Li, Jun Lu, Jie Wang, Peizhi Deng, Changjiang Meng, Haibo Tang

**Affiliations:** ^1^ Center of Clinical Pharmacology, The Third Xiangya Hospital, Central South University, Changsha, China; ^2^ Department of Cardiology, The Third Xiangya Hospital, Central South University, Changsha, China; ^3^ School of Medicine, Hunan Normal University, Changsha, China; ^4^ Department of Metabolic and Bariatric Surgery, The Third Xiangya Hospital, Central South University, Changsha, China

**Keywords:** Mendelian randomization, inflammatory cytokines, ischemic stroke, causal inference, instrumental variable (IV)

## Abstract

**Background:** Observational studies have revealed the association between some inflammatory cytokines and the occurrence of ischemic stroke, but the causal relationships remain unclear.

**Methods:** We conducted a two-sample Mendelian randomization (MR) analysis to assess the causal effects of thirty inflammatory cytokines and the risk of ischemic stroke. For exposure data, we collected genetic variants associated with inflammatory cytokines as instrumental variables (IVs) from a genome-wide association study (GWAS) meta-analysis from Finland (sample size up to 8,293). For the outcome data, we collected summary data of ischemic stroke from a large-scale GWAS meta-analysis involved 17 studies (34,217 cases and 406,111 controls). We further performed a series of sensitivity analyses as validation of primary MR results.

**Results:** According to the primary MR estimations and further sensitivity analyses, we established one robust association after Bonferroni correction: the odds ratio (95% CI) per unit change in genetically increased IL-4 was 0.84 (0.89–0.95) for ischemic stroke. The chemokine MCP3 showed a nominally significant association with ischemic stroke risk (OR: 0.93, 95% CI: 0.88–0.99, unadjusted *p* < 0.05). There was no evidence of a causal effect of other inflammatory cytokines and the risk of ischemic stroke.

**Conclusions:** Our study suggested that genetically increased IL-4 levels showed a protective effect on the risk of ischemic stroke, which provides important new insights into the potential therapeutic target for preventing ischemic stroke.

## Introduction

Stroke has become one of the leading causes of death and long-term disability in humans (D 2019 Stroke Collabora, 2021). Ischemic strokes account for approximately 70% of the incidence of stroke and have an extremely high morbidity and mortality rate. Globally, more than nine million people suffer from ischemic stroke each year, placing a vast medical burden on society (Phipps and Cronin, 2020; Powers, 2020), suggesting an urgent need to find new treatment strategies to control stroke. Ischemic stroke results from the disruption of blood supply to the brain due to various causes and the corresponding neurological deficits. In addition to the traditional risk factors such as hypertension, diabetes, smoking, alcohol consumption, and obesity, inflammatory factors play a crucial role in ischemic stroke, with both adverse and beneficial effects (Bonaventura et al., 2016).

Inflammation promotes infarct enlargement and is accountable for its resolution with implications for remodeling and repair (Lambertsen et al., 2019). This conflicting outcome may be due to genetic variation in molecules involved in inflammatory and metabolic pathways (Alfieri et al., 2020). Although experimental evidence suggests that targeting some of these inflammatory cytokines holds promise for treating ischemic stroke (Lambertsen et al., 2019), studies on the connection between circulating inflammatory cytokines and stroke risk remain scarce. Some observational studies on the role of inflammatory factors in the occurrence and recurrence of ischemic stroke have failed to find a causal relationship (Georgakis et al., 2019a).

Mendelian randomization (MR), as a widely used analytical method for causal inference, is a specific case of instrumental variable analysis. When MR assumptions are established, they could be used to identify and quantify causal relationships between exposures and outcomes of interest. This design is not susceptible to confounders and reverse causality bias (Smith and Ebrahim, 2003). In this study, we performed a two-sample MR analysis to test whether there was a causal relationship between inflammatory cytokines and the risk of ischemic stroke.

## Methods

### Study Design

**FIGURE 1:**
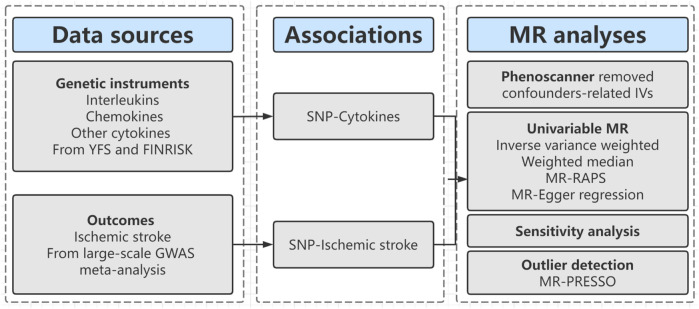
Diagram of Mendelian randomization framework in this study.

The workflow of our study is shown in Figure 1. First, we extracted genetic variants as IVs (instrumental variables) for thirty inflammatory cytokines. Second, we collected the summary data including all SNPs from the largest genome-wide association studies (GWASs) for ischemic stroke; Third, we performed two-sample MR analyses with five MR methods [e.g., inverse-variance weighted (IVW), MR-Egger regression, weighted median, MR-Robust adjusted profile score (MR-RAPS), and MR-Pleiotropy Residual Sum and Outlier (MR-PRESSO)]. Fourthly, we conducted a series of sensitivity analyses, including Cochran’s Q Test, Egger intercept test, and MR PRESSO global test to evaluate the heterogeneity and horizontal pleiotropy of MR results.

### Selection of Genetic Predictors of Inflammatory Cytokines

We obtained the genetic predictors from the most comprehensive cytokine-related GWAS meta-analysis for three independent cohorts [the Cardiovascular Risk in Young Finns Study (YFS), FINRISK 1997, and FINRISK 2002], including up to 8,293 Finnish participants (Ahola-Olli et al., 2017). The associations between genetic variants and inflammatory cytokines were adjusted for age, sex, and body mass index. We selected SNPs associated with inflammatory cytokines as IVs using a genome-wide *p*-value threshold (5 × 10^–6^, IV assumption 1, Supplementary Figure S1). A stringent condition (linkage disequilibrium threshold of *r* ^2^ < 0.001 and distance located 10,000 kb apart from each other) was set to ensure that the selection of IVs is conditionally independent of each other. *F* statistic represents the strength of the relationship between IVs and inflammatory cytokines. Generally, F > 10 may attenuate bias produced by weak IVs (Burgess and Thompson, 2011).

### Selection of Ischemic Stroke

We collected summary data of ischemic stroke from a large-scale GWAS meta-analysis containing 17 studies that involved only European participants (34,217 cases and 406,111 controls) (Malik et al., 2018). The largest available GWAS summary statistics were extracted from the MR-Base database (∼8,000,000 genetic variants) (Hemani et al., 2018). The participants had an identical genetic background (all Europeans), and to our knowledge, there was no sample overlap between the exposure and outcome GWASs.

### Statistical Analysis

#### Two-Sample Mendelian Randomization Analysis

As shown in Supplementary Figure S1, we estimated the causal association between inflammatory cytokines and ischemic stroke using a classic MR model: β_causal effect_ = *β*_ZY_/*β*_ZX_ (*β*_ZX_ and *β*_ZY_ represent the regression coefficient of SNPs on inflammatory cytokines and ischemic stroke, respectively) (Smith and Ebrahim, 2003; Zheng et al., 2017). MR analysis also relies on three IV assumptions (Supplementary Figure S1) (D 2019 Stroke Collabora, 2021): must be truly associated with inflammatory cytokines (in this study, defined as the genetic association *p* < 5 × 10^–6^) (Phipps and Cronin, 2020); not associated with confounders of inflammatory cytokines and ischemic stroke [in this study, we conducted a phenome-wide association test to assess the relationships of IVs with potential confounders such as body mass index, blood pressure, hypertension, and plasma lipid levels using PhenoScanner V2 (Kamat et al., 2019)]; and (Powers, 2020) should only be associated with ischemic stroke through inflammatory cytokines.

To evaluate the causal effects of inflammatory cytokines on the risk of ischemic stroke by combining multiple SNPs, we conducted a two-sample Mendelian randomization (Burgess et al., 2015) analysis using five primary methods, including IVW (Johnson and Uk, 2012), MR-Egger regression (Bowden et al., 2015), weighted median (Bowden et al., 2016), MR-RAPS (Zhao et al., 2020), and MR-PRESSO (Verbanck et al., 2018). The IVW is a conventional method to combine each Wald ratio estimation for multiple SNPs, with the largest statistical power among all MR methods. The weighted median estimator could provide valid estimation even when including 50% of the invalid genetic instruments. With the criterion relaxed, the MR-Egger regression provides a valid estimation even when all genetic instruments are invalid (presence of horizontal pleiotropy across SNPs). Nevertheless, it still requires the InSIDE assumption (Instrument Strength Independent of Direct Effect assumption) to be satisfied (Bowden et al., 2015). Notably, the MR-Egger regression has less power and provides wider confidence intervals. Since we used a relatively higher significant threshold (*p* < 5 × 10^–6^) to select genetic variants, we further performed the MR-RAPS to obtain MR estimations using potentially weak instruments. The MR-PRESSO regresses the SNP-outcome estimates against the SNP-exposure estimates to identify outlier SNPs and outputs a corrected MR estimate.

#### Sensitivity MR Analyses

We examined the heterogeneity of the MR results using the Cochran’s Q-test (Burgess et al., 2017) and evaluated the horizontal pleiotropy by testing whether the intercept in MR-Egger regression and MR-PRESSO global test. Once heterogeneity or horizontal pleiotropy was noted, we recomputed MR estimates after removing the outlier SNPs identified by MR-PRESSO.

Based on the 5 MR methods mentioned above, we took the IVW results as the primary MR estimates and considered the consistency of the results across other MR methods. Here, we defined the evidence for a potential causal effect when the following criteria were met (D 2019 Stroke Collabora, 2021): MR results of IVW passed the multiple comparisons adjusted *p*-value < 0.0017 (0.05/30) after Bonferroni correction (Phipps and Cronin, 2020); other MR methods showed a similar magnitude and same direction with IVW; and (Powers, 2020) there was no evidence of heterogeneity and horizontal pleiotropy (e.g., *P*_heterogeneity_, *P*_intercept_, and *p-*value for MR-PRESSO global test >0.05).

MR analysis was performed in R (version 4.0.3) with R packages “TwoSampleMR” (12), “mr-raps” (Zhao et al., 2020), and “MR-PRESSO” (Verbanck et al., 2018). *p* values were two-sided, and the statistical significance was set at the adjusted *p*-value < 0.0017.

## Results

### Participant Characteristics and Genetic Instruments

The characteristics of the participants from YFS and FINRISK and meta-analysis of GWAS for ischemic stroke are shown in Table 1. We selected 4–20 SNPs, 3–17 SNPs, 3–17 SNPs as instruments for interleukins, chemokines, and other cytokines, respectively (Supplementary Table S1), with an *F* statistic ranging from 18.7–99.7, reflecting a strong instrument strength for inflammatory cytokines.

**TABLE 1:** Characteristics of inflammatory cytokines and ischemic stroke datasets.

Exposures	Data source	SNPs	*F* Statistic	Sample size	Population
Interleukins	YFS^a^ and FINRISK^b^	4–20	18.8–52.9	3,309–8,270	European
Chemokines	YFS and FINRISK	3–17	18.7–99.7	843–8,293	European
Other cytokines	YFS and FINRISK	3–17	23.4–77.0	1,559–8,186	European
Outcome	Data source	Studies	Cases/Controls	Sample size	Population
Ischemic stroke	Meta-analysis	17	34,217/406,111	440,328	European

SNP indicates single nucleotide polymorphism; YFS, Young Finns Study; FINRISK, a large Finnish population survey on risk factors on chronic, noncommunicable diseases.

a YFS: 51% female, with the age range 34–49.

b FINRISK: 50% female, with the age range 24–74.

### Estimation of Causal Effects of Inflammatory Cytokines on Ischemic Stroke

**FIGURE 2:**
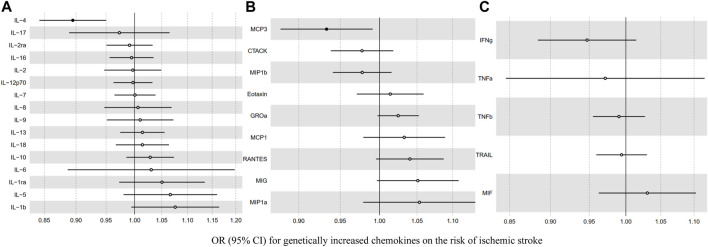
The association between genetically determined inflammatory cytokines [**(A)** interleukin **(B)** chemokine **(C)** other cytokines] and the risk of ischemic stroke.

Before we conducted MR analysis, we identified an invalid variant rs7088799, which was an instrument variant of IL-10 and IL-12p70, and also associated with body mass index, blood pressure, and hypertension (*p* < 5 × 10^–6^) using PhenoScanner V2 (Supplementary Table S2). There was no evidence that any other instrument variants violating the IV assumption 2. Table 2 and Figure 2 show the overall results of MR analysis for increased inflammatory cytokines on the risk of ischemic stroke. There was evidence to support that (*p* < 0.0017 for IVW after Bonferroni correction) genetically increased IL-4 was associated with a lower risk of ischemic stroke (OR: 0.89, 95% CI: 0.84–0.95). The results from other MR methods showed good consistency with IVW (*p* < 0.05 in weighted median and MR-RAPS, and MR-Egger showed a similar effect size). Similarly, genetically determined MCP3 showed a negative association with ischemic stroke risk (OR: 0.93, 95% CI: 0.88–0.99) at a nominally significant threshold (*p* < 0.05). However, there was little evidence to support causal relationships between other inflammatory cytokines and the risk of ischemic stroke.

**TABLE 2:** Two-sample MR estimations showing the effects of interleukins, chemokines, and other cytokines on the ischemic stroke.

Exposures	Methods	Odds ratio (95% CI)	*p*-value	Q-statistics	*P*_h_	Egger intercept	*P*_*intercept*_
Interleukins						
IL-2	MR-Egger	0.91 (1.02–1.15)	6.96E-01	14.76	9.82E-02	−4.81E-03	6.23E-01
	Inverse-variance weighted	0.95 (1.00–1.05)	9.02E-01	15.20	1.33E-01	—	—
	Weighted median	0.94 (1.00–1.06)	9.82E-01	—	—	—	—
	MR-RAPS	0.99 (0.95–1.04)	6.62E-01	—	—	—	—
IL-4	MR-Egger	0.82 (0.91–1.02)	1.54E-01	1.47	9.82E-01	−3.24E-03	7.05E-01
	Inverse-variance weighted	0.89 (0.84–0.95)	2.93E-04*	1.62	9.95E-01	—	—
	Weighted median	0.84 (0.91–0.98)	1.71E-02	—	—	—	—
	MR-RAPS	0.89 (0.84–0.95)	5.71E-04*	—	—	—	—
IL-5	MR-Egger	0.91 (0.71–1.18)	5.57E-01	2.61	2.72E-01	2.63E-02	3.35E-01
	Inverse-variance weighted	1.07 (0.98–1.16)	1.34E-01	4.68	2.03E-01	—	—
	Weighted median	1.03 (0.95–1.13)	4.76E-01	—	—	—	—
	MR-RAPS	1.06 (0.97–1.16)	2.02E-01	—	—	—	—
IL-6	MR-Egger	0.91 (0.68–1.21)	5.33E-01	20.98	8.22E-04	2.26E-02	3.57E-01
	Inverse-variance weighted	1.03 (0.89–1.20)	6.96E-01	25.43	2.85E-04	—	—
	Weighted median	1.00 (0.90–1.10)	9.41E-01	—	—	—	—
	MR-RAPS	1.01 (0.91–1.13)	8.53E-01	—	—	—	—
IL-7	MR-Egger	0.95 (0.87–1.03)	2.68E-01	11.85	3.04E-01	1.52E-02	2.24E-01
	Inverse-variance weighted	0.96 (1.00–1.04)	9.67E-01	13.89	2.43E-01	—	—
	Weighted median	0.99 (0.95–1.04)	8.69E-01	—	—	—	—
	MR-RAPS	1.00 (0.96–1.03)	8.34E-01	—	—	—	—
IL-8	MR-Egger	0.85 (0.94–1.04)	3.45E-01	0.59	7.52E-01	1.55E-02	2.53E-01
	Inverse-variance weighted	1.01 (0.95–1.07)	8.40E-01	3.25	3.64E-01	—	—
	Weighted median	0.99 (0.92–1.07)	8.67E-01	—	—	—	—
	MR-RAPS	1.01 (0.95–1.07)	8.39E-01	—	—	—	—
IL-9	MR-Egger	1.02 (0.89–1.16)	7.98E-01	0.12	9.92E-01	−2.23E-03	9.05E-01
	Inverse-variance weighted	1.01 (0.95–1.07)	7.41E-01	0.14	9.96E-01	—	—
	Weighted median	1.01 (0.94–1.08)	7.76E-01	—	—	—	—
	MR-RAPS	1.01 (0.95–1.07)	7.48E-01	—	—	—	—
IL-10^a^	MR-Egger	1.00 (0.93–1.08)	9.54E-01	27.63	6.72E-02	2.56E-02	6.72E-01
	Inverse-variance weighted	1.02 (0.98–1.06)	3.81E-01	27.91	8.53E-02	—	—
	Weighted median	1.01 (0.96–1.05)	7.73E-01	—	—	—	—
	MR-RAPS	1.02 (0.99–1.06)	1.84E-01	—	—	—	—
IL-13	MR-Egger	0.97 (0.91–1.05)	5.22E-01	4.62	3.34E-01	1.22E-01	2.76E-01
	Inverse-variance weighted	1.01 (0.97–1.06)	4.89E-01	6.51	2.63E-01	—	—
	Weighted median	1.00 (0.96–1.04)	0.95E-01	—	—	—	—
	MR-RAPS	1.01 (0.97–1.05)	5.51E-01	—	—	—	—
IL-16	MR-Egger	1.01 (0.94–1.08)	8.62E-01	21.37	6.24E-03	−4.73E-03	6.92E-01
	Inverse-variance weighted	0.99 (0.95–1.03)	7.92E-01	21.84	9.42E-03	—	—
	Weighted median	0.95 (0.98–1.02)	3.25E-01	—	—	—	—
	MR-RAPS	0.99 (0.96–1.02)	4.71E-01	—	—	—	—
IL-17	MR-Egger	0.89 (0.70–1.11)	3.30E-01	14.37	7.25E-02	1.42E-02	4.14E-01
	Inverse-variance weighted	0.89 (0.97–1.06)	5.52E-01	15.75	7.26E-02	—	—
	Weighted median	0.89 (0.99–1.10)	8.04E-01	—	—	—	—
	MR-RAPS	0.96 (0.88–1.04)	3.14E-01	—	—	—	—
IL-18	MR-Egger	1.09 (0.99–1.20)	1.20E-01	15.75	7.23E-02	−1.63E-02	1.46E-01
	Inverse-variance weighted	1.01 (0.97-1–06)	5.63E-01	20.31	2.62E-02	—	—
	Weighted median	1.01 (0.95–1.07)	7.61E-01	—	—	—	—
	MR-RAPS	1.01 (0.97–1.07)	5.19E-01	—	—	—	—
IL-1ra	MR-Egger	1.16 (0.95–1.43)	2.10E-01	9.80	8.12E-02	−1.71E-02	3.56E-01
	Inverse-variance weighted	1.05 (0.97–1.13)	2.09E-01	11.92	6.43E-02	—	—
	Weighted median	1.08 (0.99–1.17)	7.45E-02	—	—	—	—
	MR-RAPS	1.08 (0.99–1.17)	6.90E-02	—	—	—	—
IL-1b	MR-Egger	1.15 (0.98–1.35)	1.64E-01	4.81	3.13E-01	−1.24E-02	4.12E-01
	Inverse-variance weighted	1.08 (0.99–1.16)	6.98E-02	5.84	3.25E-01	—	—
	Weighted median	1.06 (0.96–1.17)	2.77E-01	—	—	—	—
	MR-RAPS	1.08 (1.00–1.18)	5.48E-02	—	—	—	—
IL-2ra	MR-Egger	1.00 (0.93–1.07)	9.45E-01	1.37	8.56E-01	−3.83E-03	7.14E-01
	Inverse-variance weighted	0.99 (0.95–1.03)	6.69E-01	1.53	9.15E-01	—	—
	Weighted median	0.99 (0.95–1.04)	7.05E-01	—	—	—	—
	MR-RAPS	0.99 (0.95–1.03)	6.77E-01	—	—	—	—
IL-12p70^a^	MR-Egger	0.99 (0.93–1.05)	7.00E-01	11.67	3.83E-01	1.92E-04	7.17E-01
	Inverse-variance weighted	1.00 (0.96–1.04)	8.74E-01	11.83	4.56E-01	—	—
	Weighted median	1.00 (0.96–1.04)	8.77E-01	—	—	—	—
	MR-RAPS	1.00 (0.96–1.03)	8.87E-01	—	—	—	—
Chemokines						
CTACK	MR-Egger	0.99 (0.91–1.08)	8.86E-01	5.92	5.52E-01	4.61E-03	6.92E-01
	Inverse-variance weighted	0.98 (0.94–1.02)	2.72E-01	6.10	6.43E-01	—	—
	Weighted median	1.01 (0.95–1.06)	7.94E-01	—	—	—	—
	MR-RAPS	0.99 (0.95–1.03)	6.37E-01	—	—	—	—
Eotaxin	MR-Egger	1.00 (0.89–1.12)	9.58E-01	15.53	4.14E-01	2.52E-03	7.71E-01
	Inverse-variance weighted	1.01 (0.97–1.06)	5.30E-01	15.62	4.85E-01	—	—
	Weighted median	0.98 (0.91–1.06)	6.58E-01	—	—	—	—
	MR-RAPS	1.01 (0.97–1.06)	6.60E-01	—	—	—	—
GROa	MR-Egger	1.01 (0.95–1.07)	8.18E-01	5.57	4.73E-01	6.91E-03	5.33E-01
	Inverse-variance weighted	1.02 (1.00–1.05)	7.70E-02	6.01	5.44E-01	—	—
	Weighted median	1.01 (0.98–1.05)	4.59E-01	—	—	—	—
	MR-RAPS	1.02 (0.99–1.05)	1.11E-01	—	—	—	—
MCP1	MR-Egger	1.04 (0.93–1.71)	4.73E-01	19.93	6.84E-02	1.83E-02	
	Inverse-variance weighted	1.03 (0.98–1.09)	2.37E-01	20.01	9.55E-02		
	Weighted median	1.04 (0.98–1.10)	2.31E-01	—	—	—	—
	MR-RAPS	1.03 (0.98–1.09)	2.36E-01	—	—	—	—
MCP3	MR-Egger	0.82 (0.69–1.01)	2.83E-01	0.78	3.81E-01	3.72E-02	3.93E-01
	Inverse-variance weighted	0.93 (0.88–0.99)	2.42E-02	2.77	2.53E-01	—	—
	Weighted median	0.97 (0.91–1.04)	4.96E-01	—	—	—	—
	MR-RAPS	0.93 (0.87–0.98)	1.62E-02	—	—	—	—
MIG	MR-Egger	1.01 (0.88–1.17)	8.59E-01	10.09	1.82E-01	7.92E-03	6.24E-01
	Inverse-variance weighted	1.05 (1.00–1.11)	6.69E-02	10.48	2.32E-01	—	—
	Weighted median	1.09 (0.97–1.23)	1.93E-01	—	—	—	—
	MR-RAPS	1.06 (1.00–1.11)	3.27E-02	—	—	—	—
MIP1a	MR-Egger	0.92 (0.76–1.11)	4.25E-01	10.25	1.13E-01	2.53E-02	1.96E-01
	Inverse-variance weighted	1.05 (0.98–1.13)	1.65E-01	14.02	5.13E-02	—	—
	Weighted median	1.08 (0.97–1.20)	1.89E-01	—	—	—	—
	MR-RAPS	1.05 (0.98–1.13)	1.35E-01	—	—	—	—
MIP1b	MR-Egger	0.99 (0.94–1.05)	7.37E-01	20.18	9.15E-02	4.08E-03	5.35E-01
	Inverse-variance weighted	0.98 (0.94–1.02)	2.47E-01	20.83	1.16E-01	—	—
	Weighted median	0.99 (0.96–1.03)	7.23E-01	—	—	—	—
	MR-RAPS	0.97 (0.93–1.01)	1.38E-01	—	—	—	—
RANTES	MR-Egger	1.05 (0.93–1.20)	4.41E-01	3.02	9.37E-01	2.87E-03	8.44E-01
	Inverse-variance weighted	1.04 (1.00–1.09)	7.88E-02	3.06	9.68E-01	—	—
	Weighted median	1.04 (1.00–1.09)	3.04E-01	—	—	—	—
	MR-RAPS	1.04 (0.97–1.13)	9.05E-02	—	—	—	—
Other cytokines						
IFNg	MR-Egger	1.03 (0.90–1.19)	6.67E-01	8.07	4.32E-01	-1.23E-02	2.14E-01
	Inverse-variance weighted	0.95 (0.88–1.01)	1.21E-01	9.96	3.53E-01	—	—
	Weighted median	0.97 (0.89–1.06)	5.70E-01	—	—	—	—
	MR-RAPS	0.96 (0.89–1.03)	2.43E-01	—	—	—	—
MIF	MR-Egger	1.02 (0.86–1.20)	8.68E-01	2.77	2.52E-01	2.93E-03	8.67E-01
	Inverse-variance weighted	1.03 (0.96–1.10)	3.88E-01	2.82	4.26E-01	—	—
	Weighted median	1.02 (0.94–1.11)	6.75E-01	—	—	—	—
	MR-RAPS	1.03 (0.96–1.11)	4.17E-01	—	—	—	—
TRAIL	MR-Egger	0.99 (0.94–1.03)	5.62E-01	19.65	1.93E-01	—	—
	Inverse-variance weighted	0.99 (0.96–1.03)	7.43E-01	20.02	2.24E-01	2.92E-03	6.03E-01
	Weighted median	0.99 (0.94–1.04)	6.20E-01	—	—	—	—
	MR-RAPS	1.00 (0.97–1.04)	8.04E-01	—	—	—	—
TNFb	MR-Egger	1.01 (0.95–1.07)	7.75E-01	0.41	8.25E-01	-7.75E-03	4.92E-01
	Inverse-variance weighted	0.99 (0.95–1.03)	6.02E-01	1.12	7.73E-01	—	—
	Weighted median	0.99 (0.95–1.03)	6.54E-01	—	—	—	—
	MR-RAPS	0.99 (0.95–1.03)	6.12E-01	—	—	—	—
TNFa	MR-Egger	0.80 (0.69–0.94)	2.25E-01	0.77	3.84E-01	3.71E-02	2.36E-01
	Inverse-variance weighted	0.97 (0.85–1.12)	6.84E-01	7.70	2.13E-02	—	—
	Weighted median	0.99 (0.90–1.08)	7.63E-01	—	—	—	—
	MR-RAPS	0.93 (0.82–1.06)	2.84E-01	—	—	—	—

MR indicates Mendelian randomization; *P*h, *P*-value for heterogeneity; RAPS, Robust adjusted profile score; IL, interleukin; CTACK, cutaneous T-cell attracting chemokine; GROa, growth-regulated oncogene-α; MCP1, monocyte chemotactic protein-1; MCP3, monocyte chemotactic protein-3; MIG, monokine induced by interferon gamma; MIP1a, macrophage inflammatory protein-1α; MIP1b, macrophage inflammatory protein-1β; RANTES, regulated on Activation, Normal T Cell 24 Expressed and Secreted; IFNg, interferon gamma; MIF, macrophage-migration inhibitory factor; TRAIL, TNF-related apoptosis-inducing ligand; TNF, tumor necrosis factor.

a SNP, rs7088799, associated with body mass index, blood pressure, and self-reported hypertension (*p* < 5 × 10^–6^) using PhenoScanner V2 (Supplementary Table 2), was removed when calculating MR results in cytokines IL-10, and IL-12p70. *p*-values in bold indicates they achieved the nominal significance (*p* < 0.05).

* *p*-values passed the Bonferroni correction tests (*p* < 0.0017).

### Validation of MR Results Using Sensitivity Analyses

We carried out a series of sensitivity analyses to evaluate the heterogeneity and potential horizontal pleiotropy (Table 2). Cochran’s Q-test showed clear evidence (*P*_h_ < 0.05) for the presence of heterogeneity for the MR results of IL-6, IL-16, and TNFα (Table 2). MR-PRESSO identified outliers and provided a corrected estimation. After removing the outlier SNPs, the recomputed MR estimations of IL-6, IL-16 were similar to the results mentioned above, and MR-PRESSO failed to provide any results for TNFα because it proxied by only three SNPs (Supplementary Table S3). The MR-Egger intercept test showed no horizontal pleiotropy (*P*_intercept_ > 0.05) for all inflammatory cytokines.

## Discussion

In this study, we performed a two-sample MR analysis to test if genetic evidence supported a causal relationship of inflammatory cytokines with the risk of ischemic stroke. Understanding the effect of inflammatory factors in ischemic stroke will help us further insights into how inflammation contributes to the initiation and progression of ischemic stroke. We found that genetically predicted IL-4 levels were negatively associated with ischemic stroke, which adds to epidemiological evidence to the role of inflammatory factor-targeted drug therapy in the prevention and treatment of ischemic stroke.

To our knowledge, there are three studies evaluating the association between partial interleukins and the risk of ischemic stroke so far (Jenny et al., 2019; Lin et al., 2020; Yuan et al., 2020). Jenny et al. reported that IL-6 was linked to an increased incidence of ischemic stroke and no significant associations of IL-8 and IL-10 with ischemic stroke risk (Jenny et al., 2019). Nevertheless, there were unmeasured confounders or misclassification of covariates that could lead to bias (Jenny et al., 2019). In addition, it was shown that IL-6 might promote early clinical deterioration of ischemic stroke, and TNF-α did not play a role in early clinical deterioration (Vila et al., 2000). Lin et al. found that IL-1ra is positively linked to cardioembolic stroke while IL-6 was negatively linked to stroke and coronary artery disease (Yuan et al., 2020). However, another study reported that genetically elevated IL-1Ra, soluble IL-6 receptor (sIL-6R), and C-reactive protein (CRP) levels are not causally associated with ischemic stroke. Our study found no statistically significant effect of IL-6 and IL-1ra on ischemic stroke. This inconsistency in results may be due to differences in sample size and different proportions of stroke subtypes in various populations (Tso et al., 2007). Besides, our study confirmed that increased IL-4 was associated with a reduced risk of ischemic stroke. The possible explanation is that the anti-inflammatory factor IL-4 acts by binding to the IL-4 receptor, enhancing the IL-4 signaling pathway, reducing the incidence of ischemic stroke, and promoting recovery after ischemic stroke (Xiong et al., 2011; Ferreira et al., 2014; Liu et al., 2016; Lively et al., 2016; Chen et al., 2020). Bis et al. reported that IL-1b promotes the ischemic stroke risk (Bis et al., 2008). However, in our study, the association between IL-1b and ischemic stroke was attenuated (OR:1.08, 95%CI: 0.99–1.16, *p* = 0.07). The lack of significance was probably explained by insufficient statistical power because we noticed that the confidence interval of IL-1b was wider than most other cytokines.

Most current studies focus on the effect of chemokines on stroke rather than ischemic stroke. Early animal experiments suggested elevated MCP3 was associated with stroke, especially in aging mice (Townshend et al., 2015). Extensive research using animal models has shown a vital role for monocyte chemotactic protein-1 (MCP1) in atherogenesis and atheroprogression (Aiello et al., 1999; Inoue et al., 2002; Lin et al., 2014). By binding to the receptor CCR2, MCP1 attracts monocytes by being upregulated under chronic inflammatory conditions. There was an observational study involving 17,180 individuals to determine the relationship between circulating levels of MCP1 and incident stroke in the general population (Georgakis et al., 2019b). It was reported that higher circulating MCP1 increases the risk of stroke. Further MR analysis supported this finding in the MEGASTROKE dataset, however, it showed no statistically significant correlation between MCP1 and ischemic stroke (OR: 1.07, 95% CI: 0.97–1.18, *p* = 0.17) in the UK Biobank (Georgakis et al., 2019c). In our study, we found no evidence to support that genetically determined MCP1 was associated with the risk of ischemic stroke. This might be partially attributable to different standards for selecting IVs and proportions of stroke subtypes in different populations.

Our study systematically evaluated the association between inflammatory cytokines and ischemic stroke risk using summary-level data from a large-scale GWAS meta-analysis. There are also several limitations to this study. Firstly, we extracted IVs using a relatively higher threshold (*p* < 5 × 10^–6^), which weak IVs may bias. However, considering both *F*-statistics and MR-RAPS results, it appears unlikely that weak IVs could have influenced our findings. Secondly, the association of inflammatory cytokines with stroke risks may not be a linear relationship, and we could not obtain the individual-level data to perform a further non-linear MR analysis. Thirdly, the population in this study is restricted to individuals of European ancestry, which means that our findings may not be extendable to other individuals of ancestry.

## Conclusion

In summary, our study supported evidence that genetically determined IL-4 levels are related to the reduced risk of ischemic stroke, suggesting that regulation and intervention of certain inflammatory factors might represent an effective strategy for the future treatment and prevention of ischemic stroke.

## Data Availability

The original contributions presented in the study are included in the article/Supplementary Material, further inquiries can be directed to the corresponding author.

## Abbreviations

CCR2, C-C Motif Chemokine Receptor 2; CI, confidence interval; CRP, C-reactive protein; GWAS, genome-wide association study; IL, Interleukin; InSIDE, Instrument Strength Independent of Direct Effect; IVs, instrumental variables; IVW, inverse-variance weighted; MCP, monocyte chemotactic protein; MR, Mendelian randomization; MR-PRESSO, MR-Pleiotropy Residual Sum and Outlier; MR-RAPS, MR-Robust adjusted profile score; OR, odds ratio; sIL-6R, soluble IL-6 receptor; SNPs, Single-nucleotide polymorphisms; TNFα, tumor necrosis factor α; YFS, Young Finns Study.
