# Supplementary material for: Inflammatory Cytokines and Risk of Ischemic Stroke: A Mendelian Randomization Study
Source: Front Pharmacol. 2022 Jan 17;12:779899. doi: 10.3389/fphar.2021.779899 (PMC8801801; doi:10.3389/fphar.2021.779899)
Supplement: Supplementary file 1 [file DataSheet2.PDF]

Table S1. Single-nucleotide polymorphisms associated with cytokines.

SNP	effect_allele	other_allele	pval	beta	se	eaf	exposure
rs10457128	G	A	5.24E-07	0.0865	0.0172	0.3608	IL10
rs10493718	C	A	7.16E-07	0.11	0.0222	0.7087	IL10
rs11206302	C	T	2.20E-06	0.1189	0.0251	0.0795	IL10
rs2086656	C	T	3.78E-06	0.0789	0.0171	0.3052	IL10
rs282258	C	T	1.00E-09	-0.0992	0.0162	0.5746	IL10
rs3025021	C	T	1.46E-06	-0.0947	0.0195	0.6322	IL10
rs35794877	C	A	1.80E-06	-0.0794	0.0167	0.508	IL10
rs41282660	G	A	3.72E-06	0.1194	0.0255	0.1322	IL10
rs4349809	G	T	5.77E-67	-0.2853	0.0165	0.4513	IL10
rs465757	G	A	1.17E-06	-0.084	0.0174	0.333	IL10
rs4741748	G	A	2.79E-06	0.0793	0.017	0.4284	IL10
rs7088799	G	T	3.23E-07	0.0852	0.0167	0.4344	IL10
rs10738760	G	A	1.02E-08	-0.0896	0.0156	0.497	IL12p70
rs13209117	G	A	5.57E-08	-0.1002	0.0186	0.7157	IL12p70
rs17229494	G	A	4.93E-06	0.1172	0.0257	0.0865	IL12p70
rs200757	C	T	4.15E-06	0.0985	0.0214	0.8767	IL12p70
rs282258	C	T	3.21E-06	-0.073	0.0156	0.5746	IL12p70
rs41282644	G	A	1.05E-06	-0.1473	0.0304	0.9364	IL12p70
rs4349809	G	T	2.56E-124	-0.3777	0.0159	0.4513	IL12p70
rs4734879	G	A	1.22E-06	-0.0918	0.0189	0.2922	IL12p70
rs7088799	G	T	6.29E-10	0.0998	0.0161	0.4344	IL12p70
rs71361173	G	T	3.06E-06	-0.111	0.0239	0.1133	IL12p70
rs72831623	G	A	2.42E-07	-0.1913	0.037	0.9473	IL12p70
rs782107	G	A	1.60E-06	-0.075	0.0156	0.4453	IL12p70
rs79121401	C	T	4.24E-06	-0.5548	0.1206	0.0189	IL12p70
rs117795020	G	A	9.86E-07	0.3522	0.0716	0.9821	IL13
rs12623722	G	A	4.19E-06	0.1185	0.0258	0.7177	IL13
rs139083458	C	T	2.81E-06	-0.9902	0.2107	0.9841	IL13
rs142167313	C	T	3.98E-07	0.313	0.0617	0.0189	IL13
rs27949	C	T	3.43E-06	0.1168	0.0252	0.3688	IL13
rs6799107	C	T	1.25E-06	0.1459	0.0301	0.2137	IL13
rs7073807	C	T	2.37E-06	-0.1682	0.0356	0.8827	IL13
rs75995699	G	A	2.64E-06	-0.3319	0.0698	0.9761	IL13
rs9472168	G	A	1.08E-65	-0.4244	0.0248	0.4493	IL13
rs116135478	G	A	3.66E-06	-0.8206	0.166	0.0179	IL16
rs117217798	C	T	4.15E-06	0.2036	0.0444	0.9314	IL16
rs117916513	G	A	3.79E-07	0.502	0.0986	0.9791	IL16
rs1255143	C	T	7.10E-08	-0.1306	0.0242	0.4344	IL16
rs12765671	G	A	4.84E-06	0.6023	0.1318	0.9831	IL16
rs144691581	G	A	4.20E-07	-0.4882	0.0967	0.9851	IL16
rs1801020	G	A	4.53E-10	-0.1733	0.0272	0.7753	IL16
rs4253283	C	T	1.75E-08	-0.146	0.0262	0.6998	IL16
rs4513633	C	A	7.44E-07	0.2239	0.0453	0.1243	IL16
rs4778636	G	A	1.11E-30	0.7272	0.0633	0.9254	IL16
rs9706053	C	T	7.01E-07	-0.4582	0.0932	0.9841	IL16
rs117029961	G	A	4.94E-06	-0.4585	0.1015	0.9891	IL17
rs117556572	C	T	3.28E-06	0.5102	0.1099	0.9891	IL17
rs1530455	C	T	4.87E-10	-0.108	0.0173	0.5915	IL17
rs17106604	C	T	6.37E-07	-0.1129	0.0225	0.8757	IL17
rs17282552	C	T	8.21E-07	0.2001	0.0405	0.0268	IL17
rs184080173	C	T	4.19E-07	-0.2384	0.0471	0.0626	IL17
rs187475560	C	T	3.29E-06	0.2434	0.052	0.9881	IL17
rs34120897	C	A	3.63E-06	0.1055	0.0232	0.0974	IL17
rs62191444	G	T	4.22E-06	0.1136	0.0247	0.834	IL17
rs78296352	G	T	4.27E-06	-0.3027	0.0646	0.9712	IL17

rs78612928	C	T	2.62E-06	-0.1037	0.0222	0.1968	IL17
rs79462337	G	T	1.41E-06	-0.2097	0.0435	0.0199	IL17
rs10414578	C	T	4.16E-07	0.1771	0.035	0.8787	IL18
rs115267715	C	T	1.72E-08	-0.4508	0.08	0.9821	IL18
rs116383510	C	A	3.00E-07	0.5426	0.1056	0.0199	IL18
rs11700536	C	T	4.21E-06	-0.1156	0.025	0.6382	IL18
rs117266781	C	T	3.15E-06	-0.6841	0.1468	0.9891	IL18
rs144841621	C	T	3.81E-06	-0.518	0.1141	0.9891	IL18
rs17229943	C	A	1.62E-11	0.312	0.0463	0.0497	IL18
rs1852105	C	T	4.32E-06	-0.3036	0.0661	0.9513	IL18
rs1979967	C	T	9.45E-07	-0.1402	0.0286	0.7406	IL18
rs2729385	G	A	3.79E-06	-0.1231	0.0262	0.675	IL18
rs385076	C	T	1.66E-22	0.2432	0.0248	0.6471	IL18
rs4482818	G	A	1.45E-07	-0.1286	0.0244	0.3588	IL18
rs658805	G	A	4.94E-07	-0.1226	0.0244	0.6869	IL18
rs71478720	C	T	3.71E-22	0.2669	0.0276	0.7823	IL18
rs78623212	C	T	6.71E-07	-0.8705	0.1778	0.9761	IL18
rs78716465	G	A	1.63E-06	-0.3265	0.0682	0.9622	IL18
rs115242021	C	A	5.07E-07	-0.2326	0.0414	0.9185	IL1b
rs143319329	C	T	2.00E-06	-0.2801	0.0715	0.9761	IL1b
rs1942793	G	T	4.98E-06	-0.0717	0.0187	0.5258	IL1b
rs61335305	C	A	1.90E-06	-0.2966	0.0724	0.9891	IL1b
rs62015704	G	A	2.09E-06	-0.1082	0.0283	0.1213	IL1b
rs9898641	C	T	3.59E-06	0.2032	0.0454	0.3598	IL1b
rs1054402	C	T	1.13E-06	-0.1311	0.027	0.7336	IL1ra
rs11627423	C	A	2.12E-06	-0.1171	0.0247	0.3897	IL1ra
rs12121840	C	T	2.43E-06	-0.2692	0.0571	0.9155	IL1ra
rs139005642	AAAAG	A	1.97E-06	-0.1315	0.0277	0.1471	IL1ra
rs2809154	C	T	3.74E-06	0.1786	0.0388	0.838	IL1ra
rs56134659	G	A	2.44E-06	0.1117	0.0237	0.4742	IL1ra
rs61335305	C	A	1.00E-06	-0.4453	0.0908	0.9891	IL1ra
rs9623661	C	T	3.86E-06	0.1966	0.0426	0.9076	IL1ra
rs10903540	G	A	4.19E-06	0.1581	0.0343	0.0338	IL2
rs12051139	C	T	4.76E-06	0.1131	0.0247	0.4006	IL2
rs13412535	G	A	1.18E-07	-0.1764	0.0332	0.7744	IL2
rs1534019	C	T	1.58E-06	0.1187	0.0248	0.5557	IL2
rs170117	C	T	3.87E-06	0.1617	0.0349	0.8797	IL2
rs2807544	G	A	3.41E-06	-0.1175	0.0253	0.5656	IL2
rs4634519	G	A	2.77E-06	0.1261	0.0269	0.3121	IL2
rs61335305	C	A	7.32E-07	-0.4514	0.0918	0.9891	IL2
rs62124990	G	T	3.22E-06	0.6961	0.1495	0.9841	IL2
rs7615304	G	A	1.21E-06	0.1172	0.0242	0.6511	IL2
rs80336398	C	T	2.82E-06	-0.4001	0.0858	0.0209	IL2
rs11241559	G	T	2.00E-06	0.1264	0.0266	0.7813	IL2ra
rs115360066	G	A	8.06E-07	-0.1867	0.0379	0.1103	IL2ra
rs117244812	G	A	2.10E-06	0.7064	0.1488	0.9861	IL2ra
rs12722497	C	A	1.57E-38	-0.6279	0.0485	0.8559	IL2ra
rs185231391	C	T	1.47E-06	-0.8503	0.1809	0.0149	IL2ra
rs4733117	C	A	2.63E-06	-0.1369	0.0292	0.164	IL2ra
rs61705228	C	T	3.99E-06	-0.3303	0.0716	0.9632	IL2ra
rs62135626	C	T	1.99E-06	0.1661	0.035	0.8847	IL2ra
rs10512267	C	T	2.94E-07	0.0824	0.0161	0.33	IL4
rs116705532	G	T	1.76E-06	0.4678	0.0978	0.0169	IL4
rs117146485	C	T	2.71E-06	0.2924	0.0629	0.0149	IL4
rs17713451	G	A	4.97E-07	-0.1274	0.0253	0.835	IL4
rs2332982	G	A	3.20E-07	-0.1118	0.0219	0.1421	IL4
rs6765768	G	A	2.00E-06	-0.0795	0.0167	0.3917	IL4
rs73023729	G	A	9.03E-07	0.1796	0.0366	0.9771	IL4

rs7613691	G	A	4.05E-06	-0.1775	0.0384	0.0527	IL4
rs79597994	C	T	4.32E-06	0.5831	0.127	0.9722	IL4
rs9508291	C	T	3.03E-06	0.1676	0.0359	0.0646	IL4
rs9941733	G	A	6.88E-07	-0.114	0.0229	0.175	IL4
rs11680908	G	A	2.03E-06	-0.2634	0.0554	0.0547	IL5
rs6737109	C	T	2.40E-06	-0.116	0.0247	0.4254	IL5
rs72831687	G	A	1.69E-06	0.5239	0.1109	0.9831	IL5
rs73040130	C	T	6.00E-07	-0.2638	0.0529	0.0646	IL5
rs7767396	G	A	7.69E-10	-0.1515	0.0246	0.4513	IL5
rs114373846	C	T	3.32E-06	-0.422	0.0904	0.9871	IL6
rs12024374	G	A	3.35E-06	-0.1096	0.0236	0.8837	IL6
rs1333040	C	T	3.17E-06	-0.0738	0.0158	0.4264	IL6
rs13412535	G	A	7.34E-08	0.1164	0.0215	0.7744	IL6
rs72831623	G	A	1.08E-07	-0.1973	0.0372	0.9473	IL6
rs73273528	C	T	9.58E-07	-0.2672	0.0553	0.9662	IL6
rs76856708	C	T	2.61E-06	-0.3289	0.07	0.0378	IL6
rs117509142	C	T	1.99E-06	0.327	0.0688	0.0567	IL7
rs141425475	C	T	2.53E-06	0.4781	0.1016	0.0288	IL7
rs144701438	G	A	9.75E-07	0.4819	0.0989	0.9462	IL7
rs17091524	C	T	1.91E-06	-0.4924	0.1013	0.0427	IL7
rs218260	C	T	2.93E-06	0.1337	0.0286	0.7883	IL7
rs28793375	C	T	4.46E-06	-0.1638	0.0361	0.833	IL7
rs4320361	G	T	6.87E-39	0.3245	0.0249	0.5477	IL7
rs62006410	C	T	3.39E-07	0.1557	0.0303	0.7853	IL7
rs75904417	C	A	1.16E-06	0.1698	0.0349	0.1412	IL7
rs77318030	C	T	3.74E-06	0.2921	0.0632	0.0517	IL7
rs77981494	C	T	1.07E-06	0.5178	0.1064	0.0159	IL7
rs78346957	G	A	4.51E-06	-0.4588	0.1007	0.9771	IL7
rs11634944	C	T	1.29E-06	0.1214	0.0252	0.3469	IL8
rs12075	G	A	3.88E-07	-0.12	0.0236	0.3976	IL8
rs141926526	C	A	2.57E-06	0.6149	0.1308	0.0477	IL8
rs2673604	C	A	7.02E-07	0.1266	0.0255	0.2992	IL8
rs117935362	G	A	4.85E-06	0.5107	0.1114	0.9851	IL9
rs41294750	C	T	2.37E-06	-0.3514	0.0748	0.9722	IL9
rs4880409	C	T	3.50E-06	0.3355	0.0723	0.0278	IL9
rs61867538	C	T	3.93E-06	-0.3566	0.0774	0.9732	IL9
rs7232268	G	A	2.53E-06	-0.2759	0.0587	0.9523	IL9
rs7242404	G	A	3.27E-06	0.1228	0.0264	0.7017	IL9
rs76963786	C	T	4.50E-07	0.2865	0.0557	0.9135	IL9
rs10809307	C	T	3.64E-06	-0.1305	0.0282	0.6958	IL10
rs113831257	G	A	2.53E-08	-0.3592	0.0644	0.9583	IL10
rs11626201	C	A	1.93E-06	-0.1162	0.0245	0.3658	IL10
rs143799975	G	A	1.00E-06	0.7984	0.1637	0.0129	IL10
rs181236764	C	T	3.22E-06	-0.4504	0.0964	0.9573	IL10
rs34383175	C	T	1.51E-06	0.3153	0.0657	0.9732	IL10
rs397816	C	T	7.90E-07	-0.1237	0.0249	0.4264	IL10
rs75970138	G	A	1.53E-06	0.485	0.104	0.9871	IL10
rs7645625	G	T	4.41E-06	0.1086	0.0237	0.4324	IL10
rs79848609	C	A	8.75E-07	-0.2603	0.0537	0.0318	IL10
rs8112909	G	A	1.94E-06	0.1426	0.0299	0.1918	IL10
rs9450351	C	T	1.48E-08	0.2768	0.0489	0.0616	IL10
rs10835056	G	T	2.60E-06	-0.1194	0.0254	0.7555	MIP1a
rs116615337	G	A	4.82E-06	-0.1278	0.0279	0.2913	MIP1a
rs12690897	G	A	2.11E-06	-0.1248	0.0262	0.7187	MIP1a
rs184154340	G	A	1.86E-06	-0.331	0.0693	0.9592	MIP1a
rs34771762	G	A	2.13E-06	-0.249	0.0523	0.0736	MIP1a
rs57786342	G	A	4.06E-06	-0.1314	0.0285	0.7843	MIP1a
rs60198979	G	A	2.62E-06	0.2146	0.0458	0.9135	MIP1a

rs6900267	C	A	2.89E-06	0.2429	0.0519	0.0746	MIP1a
rs7232268	G	A	2.55E-06	-0.2821	0.0599	0.9523	MIP1a
rs113010081	C	T	3.85E-140	0.5954	0.0236	0.1083	MIP1b
rs113877493	C	T	1.62E-173	0.6124	0.0218	0.8777	MIP1b
rs116237296	G	A	7.23E-07	-0.5437	0.1115	0.9841	MIP1b
rs117453826	G	A	5.07E-22	0.5774	0.0593	0.0159	MIP1b
rs12490293	C	T	2.12E-07	-0.0866	0.0168	0.3917	MIP1b
rs141102180	G	T	1.08E-16	-0.3225	0.0393	0.9821	MIP1b
rs1437220	C	T	3.53E-06	-0.1478	0.0315	0.0497	MIP1b
rs1564708	C	T	2.87E-20	0.1744	0.0188	0.7296	MIP1b
rs17138331	G	A	2.26E-06	0.1391	0.0295	0.0944	MIP1b
rs2411161	C	T	3.14E-06	-0.1714	0.0367	0.0507	MIP1b
rs281749	C	T	3.17E-06	-0.0799	0.0171	0.7083	MIP1b
rs3760440	G	A	2.75E-14	-0.1236	0.0162	0.331	MIP1b
rs62242409	C	T	3.43E-11	-0.1282	0.0193	0.7425	MIP1b
rs72791296	C	T	3.78E-07	-0.2369	0.0466	0.9414	MIP1b
rs72799710	C	T	3.21E-06	0.1014	0.0218	0.834	MIP1b
rs74810984	C	T	1.96E-06	-0.2206	0.0474	0.0219	MIP1b
rs76582507	G	A	3.26E-06	-0.3175	0.0677	0.9732	MIP1b
rs76583883	G	T	4.99E-06	0.2317	0.0511	0.9682	MIP1b
rs76776296	G	A	5.55E-07	-0.2997	0.0598	0.0368	MIP1b
rs9793308	G	A	2.52E-06	-0.0835	0.0178	0.3718	MIP1b
rs9850846	G	A	4.48E-06	-0.072	0.0157	0.4443	MIP1b
rs11087905	C	A	5.48E-07	-0.0941	0.0189	0.6581	Eotaxin
rs112347425	C	T	8.65E-09	-0.158	0.0277	0.8926	Eotaxin
rs12075	G	A	1.33E-26	-0.1671	0.0156	0.3976	Eotaxin
rs138125931	C	T	3.59E-07	-0.1292	0.0254	0.8678	Eotaxin
rs1476670	C	A	3.51E-06	0.1007	0.0217	0.7932	Eotaxin
rs2024050	G	A	1.10E-08	-0.1728	0.0303	0.8996	Eotaxin
rs2210755	C	T	4.85E-06	0.1104	0.0242	0.0805	Eotaxin
rs2211994	C	T	6.08E-07	-0.0885	0.0177	0.7495	Eotaxin
rs2228467	C	T	2.27E-46	0.4163	0.0292	0.0696	Eotaxin
rs2419841	C	T	4.98E-06	0.1277	0.0279	0.1193	Eotaxin
rs5746492	G	A	3.96E-06	-0.0954	0.0207	0.1809	Eotaxin
rs5754733	C	A	1.06E-06	0.1042	0.0214	0.2117	Eotaxin
rs59808887	C	T	2.91E-06	0.1673	0.0358	0.9225	Eotaxin
rs75426604	C	A	2.53E-06	0.1366	0.0291	0.8688	Eotaxin
rs79722574	C	T	1.06E-06	0.1113	0.0228	0.84	Eotaxin
rs80341932	G	A	6.69E-07	-0.1016	0.0205	0.2922	Eotaxin
rs9317045	C	A	5.82E-07	-0.1182	0.0237	0.1461	Eotaxin
rs10145849	G	A	3.41E-06	0.0755	0.0162	0.6511	MCP1
rs10744620	C	T	9.91E-07	-0.0788	0.0161	0.6392	MCP1
rs111995966	G	T	2.53E-06	-0.1452	0.031	0.0258	MCP1
rs12073356	G	A	4.17E-06	0.1426	0.0311	0.9145	MCP1
rs12075	G	A	1.44E-44	-0.2185	0.0155	0.3976	MCP1
rs146522229	C	T	3.56E-07	0.5976	0.1177	0.9761	MCP1
rs2036297	G	A	1.09E-13	-0.119	0.016	0.6571	MCP1
rs2288370	C	T	2.25E-10	0.1031	0.0163	0.3956	MCP1
rs2712431	C	A	4.76E-06	0.0787	0.0172	0.2853	MCP1
rs56212190	C	T	9.85E-07	-0.181	0.0373	0.9453	MCP1
rs7197349	G	A	2.62E-06	-0.0968	0.0206	0.1372	MCP1
rs7517040	G	A	2.44E-07	0.0987	0.0191	0.7177	MCP1
rs7632755	G	A	1.18E-20	-0.2938	0.0316	0.9195	MCP1
rs9317045	C	A	1.52E-06	-0.1134	0.0236	0.1461	MCP1
rs10892381	C	T	3.56E-07	-0.2412	0.0476	0.3002	MCP3
rs62492260	G	T	1.54E-06	0.2788	0.058	0.8509	MCP3
rs73669117	G	A	2.56E-06	0.6238	0.131	0.0149	MCP3
rs111607343	G	A	2.83E-06	0.521	0.1119	0.9622	MIG

rs11177248	G	A	4.45E-06	-0.3073	0.067	0.9394	MIG
rs112337562	G	T	2.98E-06	0.37	0.0796	0.0169	MIG
rs112861654	G	A	1.81E-07	0.2765	0.0529	0.0865	MIG
rs117831247	C	T	2.16E-06	0.8334	0.1754	0.9811	MIG
rs139010077	C	T	3.55E-06	-0.4322	0.095	0.9891	MIG
rs1796086	C	T	2.23E-07	0.2096	0.0403	0.0944	MIG
rs41272086	G	A	7.43E-08	0.2226	0.0415	0.9145	MIG
rs55876513	G	T	8.23E-11	-0.166	0.0255	0.2475	MIG
rs5752128	C	T	4.34E-06	0.1685	0.0369	0.0954	MIG
rs62562991	G	A	8.40E-07	-0.6236	0.126	0.9801	MIG
rs6679677	C	A	8.86E-07	-0.162	0.0329	0.9085	MIG
rs77086208	C	T	3.83E-06	-0.3226	0.0698	0.9811	MIG
rs816960	C	T	5.01E-07	0.1224	0.0244	0.7406	MIG
rs116303454	G	A	3.27E-06	-0.383	0.0816	0.9712	CTACK
rs135564	G	A	2.43E-12	0.1893	0.0268	0.7406	CTACK
rs145902143	G	A	1.03E-06	0.2838	0.0581	0.0378	CTACK
rs17321950	C	T	3.46E-06	-0.5262	0.1122	0.0278	CTACK
rs2070074	G	A	1.79E-32	-0.4467	0.0374	0.1024	CTACK
rs3766110	C	A	3.86E-06	0.1287	0.0278	0.2326	CTACK
rs55764737	C	T	4.62E-08	-0.5313	0.0972	0.0229	CTACK
rs57338032	G	A	6.23E-07	-0.1583	0.0317	0.1799	CTACK
rs7333764	C	T	2.85E-06	-0.2773	0.0593	0.9732	CTACK
rs76395525	G	A	9.55E-07	-0.5277	0.1083	0.9851	CTACK
rs112072646	G	A	6.48E-07	-0.4286	0.0862	0.9692	RANTES
rs147509526	C	T	6.93E-07	0.358	0.0717	0.9871	RANTES
rs2251660	C	A	3.83E-07	-0.1829	0.0359	0.1451	RANTES
rs4940620	G	A	3.54E-06	0.2494	0.054	0.0636	RANTES
rs62438851	G	A	2.33E-06	0.1957	0.0414	0.1352	RANTES
rs7000423	C	T	1.82E-07	0.1318	0.0253	0.674	RANTES
rs72793342	G	A	1.48E-06	0.1487	0.0308	0.7922	RANTES
rs74472919	C	T	3.97E-08	-0.3313	0.0605	0.9811	RANTES
rs75613039	C	T	4.81E-06	-0.37	0.081	0.9692	RANTES
rs818452	C	T	2.36E-06	-0.2381	0.0505	0.9484	RANTES
rs1113500	G	T	1.57E-06	-0.1174	0.0244	0.3698	GROa
rs118158560	G	A	3.42E-06	-0.2703	0.0594	0.9374	GROa
rs12075	G	A	1.24E-55	-0.3751	0.0237	0.3976	GROa
rs140734053	G	A	3.58E-06	-0.7257	0.1561	0.9751	GROa
rs185768063	G	A	1.46E-07	-0.3998	0.076	0.0139	GROa
rs188345231	C	T	4.34E-06	-0.623	0.1323	0.9881	GROa
rs508977	G	T	7.56E-42	0.3802	0.028	0.2336	GROa
rs62024303	G	A	4.41E-06	0.3053	0.0666	0.0457	GROa
rs78653452	G	T	1.21E-06	0.7362	0.1558	0.9881	GROa
rs10474392	G	A	1.24E-06	-0.0962	0.0178	0.7306	SDF1a
rs12407262	G	A	3.99E-06	-0.1179	0.0266	0.8658	SDF1a
rs13400104	G	A	4.53E-06	0.0647	0.0189	0.1799	SDF1a
rs139840550	G	A	3.79E-06	-0.1834	0.0549	0.9523	SDF1a
rs149893336	G	A	4.52E-06	0.5034	0.1081	0.0199	SDF1a
rs16872470	G	A	2.81E-07	-0.5319	0.1025	0.0129	SDF1a
rs4581824	G	T	3.05E-06	0.0701	0.0173	0.3111	SDF1a
rs482700	G	A	1.57E-06	0.0893	0.0203	0.2863	SDF1a
rs67689854	C	A	3.07E-06	0.0681	0.0195	0.8847	SDF1a
rs10834997	G	A	1.33E-06	0.1247	0.0258	0.3598	TNFa
rs111332265	G	A	6.63E-07	0.3766	0.0754	0.0716	TNFa
rs8121916	C	A	2.72E-06	-0.1306	0.0278	0.7744	TNFa
rs10925040	C	T	2.67E-06	-0.1755	0.0373	0.6113	TNFb
rs753274	C	T	2.77E-06	0.1736	0.0371	0.4642	TNFb
rs7629875	G	A	1.37E-06	-0.3766	0.0774	0.0577	TNFb
rs78296352	G	T	4.76E-21	-1.2215	0.1366	0.9712	TNFb

rs11618126	G	A	1.46E-06	-0.8908	0.1914	0.0149	TRAIL
rs11657269	G	A	4.78E-06	-0.1188	0.026	0.1193	TRAIL
rs11699445	G	T	3.27E-06	-0.0746	0.0161	0.3867	TRAIL
rs13115587	C	A	3.10E-06	-0.1019	0.0218	0.9165	TRAIL
rs13185784	G	A	3.90E-06	-0.0846	0.0183	0.7097	TRAIL
rs13278062	G	T	3.57E-07	-0.0801	0.0157	0.497	TRAIL
rs141603697	C	T	6.61E-22	-0.7202	0.0741	0.9871	TRAIL
rs146783010	G	A	4.83E-06	0.6016	0.135	0.0109	TRAIL
rs148051545	C	T	3.86E-06	0.3921	0.0848	0.9871	TRAIL
rs193112415	C	T	2.15E-62	1.0421	0.0623	0.0139	TRAIL
rs57396456	C	T	1.25E-27	0.5626	0.0518	0.0338	TRAIL
rs62093514	C	T	6.86E-82	-1.0618	0.0552	0.9742	TRAIL
rs6878823	C	T	3.48E-06	0.0879	0.0189	0.7724	TRAIL
rs73039026	C	A	2.02E-06	0.2999	0.0635	0.0159	TRAIL
rs74488044	G	A	2.61E-25	-0.3489	0.0335	0.9404	TRAIL
rs747324	C	T	1.61E-06	0.0855	0.0178	0.6412	TRAIL
rs74778900	C	T	2.59E-28	-0.5906	0.0532	0.9861	TRAIL
rs75928541	G	A	4.24E-06	-0.275	0.0593	0.9831	TRAIL
rs79287178	G	A	9.12E-25	0.4317	0.0421	0.9742	TRAIL
rs113218956	G	A	2.26E-06	0.8948	0.1879	0.9891	MIF
rs118055855	C	T	4.13E-06	-0.6907	0.15	0.0129	MIF
rs12594190	G	A	3.70E-07	-0.1355	0.0267	0.2465	MIF
rs13142904	C	T	2.56E-07	0.223	0.0425	0.9314	MIF
rs141009259	C	T	2.47E-06	0.6178	0.1322	0.0129	MIF
rs5751777	C	T	7.09E-08	0.1342	0.0249	0.3887	MIF
rs78098071	C	T	1.78E-07	0.4867	0.0918	0.0189	MIF
rs112783231	G	A	1.96E-06	0.2408	0.0511	0.9712	IFNg
rs113600793	C	A	8.95E-07	-0.1829	0.0373	0.9602	IFNg
rs115729819	G	A	1.38E-06	-0.2484	0.0515	0.0209	IFNg
rs11843756	G	T	3.09E-06	-0.184	0.0393	0.0288	IFNg
rs12420286	C	T	2.08E-06	-0.2376	0.0501	0.0288	IFNg
rs1867282	C	T	3.15E-06	-0.0774	0.0166	0.674	IFNg
rs2073438	G	A	1.68E-06	-0.0898	0.0188	0.7286	IFNg
rs4730203	G	A	1.32E-06	0.0983	0.0203	0.8618	IFNg
rs6902031	G	A	1.41E-06	0.1166	0.0242	0.8877	IFNg
rs7073753	C	T	8.39E-07	-0.0821	0.0167	0.5666	IFNg
rs74148555	C	T	2.64E-06	0.3732	0.0774	0.9473	IFNg
rs78296352	G	T	1.38E-07	-0.343	0.0652	0.9712	IFNg
