# Supplementary material for: Inflammatory Cytokines and Risk of Ischemic Stroke: A Mendelian Randomization Study
Source: Front Pharmacol. 2022 Jan 17;12:779899. doi: 10.3389/fphar.2021.779899 (PMC8801801; doi:10.3389/fphar.2021.779899)
Supplement: Supplementary file 2 [file DataSheet4.docx]

**Table S3. MR-PRESSO estimations for IL-6, IL-16, and TNFα.**

| **Exposures** | **Outliers** | **Raw OR (95% CI)** | ***P*-value** | **Corrected OR (95% CI)** | ***P*-value** | **Global test** |
| --- | --- | --- | --- | --- | --- | --- |
| IL-6 | rs1333040 | 1.03 (0.89-1.20) | 7.10E-01 | 0.97 (0.90-1.05) | 5.06E-01 | 4.00E-03 |
| IL-16 | rs12765671 | 0.99 (0.96-1.04) | 7.98E-01 | 0.99 (0.95-1.02) | 5.07E-01 | 3.80E-02 |
| TNFα* | NA | NA | NA | NA | NA | NA |

***** Not enough instrumental variables (3 SNPs).
