# Supplementary material for: Inflammatory Cytokines and Risk of Ischemic Stroke: A Mendelian Randomization Study
Source: Front Pharmacol. 2022 Jan 17;12:779899. doi: 10.3389/fphar.2021.779899 (PMC8801801; doi:10.3389/fphar.2021.779899)
Supplement: Supplementary file 3 [file DataSheet3.PDF]

Table S2. Phenome-wide association test for instrumental variables using PhenoScanner V2.

snp	a1	a2	trait	beta	se	p
rs10457128	A	G	Interleukin 10 levels	-0.0865	0.01721	5.00E-07
rs10457128	A	G	Hair or balding pattern: pattern 4	0.0112	0.001448	1.03E-14
rs10457128	A	G	Relative age of first facial hair	-0.01492	0.001651	1.57E-19
rs10493718	A	C	Interleukin 10 levels	-0.11	0.02217	7.00E-07
rs11206302	C	T	Interleukin 10 levels	0.1189	0.02501	2.00E-06
rs2086656	C	T	Interleukin 10 levels	0.0789	0.01711	4.00E-06
rs282258	C	T	Interleukin 10 levels	0.0992	0.01624	1.00E-09
rs282258	C	T	Interleukin 12p70 levels	0.073	0.01563	3.00E-06
rs3025021	C	T	Interleukin 10 levels	0.0947	0.01936	1.00E-06
rs41282660	A	G	Mean corpuscular volume	0.0255	0.005496	3.48E-06
rs4349809	G	T	Interleukin 10 levels	0.2853	0.0165	6.00E-67
rs4349809	G	T	Interleukin 12p70 levels	0.3777	0.01593	3.00E-124
rs465757	A	G	Interleukin 10 levels	-0.084	0.01717	1.00E-06
rs7088799	G	T	Basophil percentage of granulocytes	0.01884	0.003555	1.16E-07
rs7088799	G	T	Granulocyte count	-0.03503	0.003643	6.87E-22
rs7088799	G	T	Granulocyte percentage of myeloid white cells	-0.02562	0.003625	1.58E-12
rs7088799	G	T	Lymphocyte percentage of white cells	0.01782	0.003614	8.23E-07
rs7088799	G	T	Mean platelet volume	-0.1322	0.003665	5.14E-285
rs7088799	G	T	Monocyte percentage of white cells	0.02105	0.003615	5.77E-09
rs7088799	G	T	Myeloid white cell count	-0.03397	0.003652	1.38E-20
rs7088799	G	T	Neutrophil count	-0.03616	0.003633	2.47E-23
rs7088799	G	T	Neutrophil percentage of white cells	-0.02388	0.00362	4.19E-11
rs7088799	G	T	Platelet count	0.0768	0.003698	8.75E-96
rs7088799	G	T	Platelet distribution width	-0.0547	0.003645	6.55E-51
rs7088799	G	T	Plateletcrit	0.0194	0.00371	1.71E-07
rs7088799	G	T	Sum basophil neutrophil counts	-0.0355	0.00364	1.77E-22
rs7088799	G	T	Sum neutrophil eosinophil counts	-0.03567	0.003637	1.04E-22
rs7088799	G	T	White blood cell count	-0.03342	0.003636	3.92E-20
rs7088799	G	T	Interleukin 10 levels	0.0852	0.01663	3.00E-07
rs7088799	G	T	Age at first live birth	0.01817	0.003996	5.41E-06
rs7088799	G	T	Age completed full time education	0.01488	0.002461	1.49E-09
rs7088799	G	T	Alcohol usually taken with meals	0.008371	0.001574	1.04E-07
rs7088799	G	T	Average weekly beer plus cider intake	-0.01214	0.002032	2.34E-09
rs7088799	G	T	Average weekly red wine intake	0.01187	0.002445	1.21E-06
rs7088799	G	T	Body mass index	-0.01392	0.002439	1.15E-08
rs7088799	G	T	Diastolic blood pressure	-0.01604	0.002498	1.34E-10

rs7088799	G	T	Height	0.009467	0.001752	6.51E-08
rs7088799	G	T	Impedance of arm left	0.01292	0.001741	1.18E-13
rs7088799	G	T	Impedance of arm right	0.01342	0.001733	9.94E-15
rs7088799	G	T	Impedance of whole body	0.01109	0.00188	3.67E-09
rs7088799	G	T	Leg fat percentage left	-0.007538	0.001541	1.01E-06
rs7088799	G	T	Leg fat percentage right	-0.007567	0.001561	1.26E-06
rs7088799	G	T	Mineral and other dietary supplements: zinc	0.002388	0.0004798	6.49E-07
rs7088799	G	T	Qualifications: A levels or as levels or equivalent	0.006394	0.001108	7.88E-09
rs7088799	G	T	Qualifications: college or university degree	0.005683	0.001156	8.80E-07
rs7088799	G	T	Qualifications: none	-0.007234	0.000937	1.16E-14
rs7088799	G	T	Self-reported hypertension	-0.005588	0.001082	2.39E-07
rs7088799	G	T	Sodium in urine	-0.01247	0.002401	2.03E-07
rs7088799	G	T	Usual walking pace	0.008633	0.001506	9.99E-09
rs7088799	G	T	Vascular or heart problems diagnosed by doctor: high bloo	-0.005631	0.001097	2.82E-07
rs7088799	G	T	Vascular or heart problems diagnosed by doctor: none of th	0.005425	0.001126	1.46E-06
rs7088799	G	T	Alkaline phosphatase	0.01	NA	8.08E-06
rs7088799	G	T	Years of educational attainment in males	0.022	0.004	5.79E-09
rs7088799	G	T	Years of educational attainment	0.014	0.003	9.40E-09
rs10738760	A	G	Serum vascular endothelial growth factor VEGF	NA	NA	3.78E-47
rs10738760	A	G	Vascular endothelial growth factor levels	NA	NA	1.00E-39
rs10738760	A	G	Cause of death: chronic lymphocytic leukaemia	-0.005262	0.00117	6.93E-06
rs10738760	A	G	Vascular endothelial growth factor a	NA	NA	1.00E-39
rs17229494	A	G	Interleukin 12p70 levels	-0.1172	0.02567	5.00E-06
rs282258	C	T	Interleukin 10 levels	0.0992	0.01624	1.00E-09
rs282258	C	T	Interleukin 12p70 levels	0.073	0.01563	3.00E-06
rs41282644	A	G	Self-reported venous thromboembolic disease	0.0004376	8.24E-05	1.08E-07
rs4349809	G	T	Interleukin 10 levels	0.2853	0.0165	6.00E-67
rs4349809	G	T	Interleukin 12p70 levels	0.3777	0.01593	3.00E-124
rs4734879	A	G	Basophil count	0.02319	0.003919	3.29E-09
rs4734879	A	G	Granulocyte count	0.02124	0.004021	1.27E-07
rs4734879	A	G	Mean platelet volume	-0.04808	0.004059	2.31E-32
rs4734879	A	G	Myeloid white cell count	0.02186	0.00403	5.87E-08
rs4734879	A	G	Neutrophil count	0.01963	0.00401	9.80E-07
rs4734879	A	G	Platelet count	0.06957	0.004087	5.80E-65
rs4734879	A	G	Platelet distribution width	-0.06019	0.004032	2.13E-50
rs4734879	A	G	Plateletcrit	0.05318	0.004104	2.08E-38
rs4734879	A	G	Sum basophil neutrophil counts	0.02042	0.004017	3.69E-07
rs4734879	A	G	Sum neutrophil eosinophil counts	0.02075	0.004014	2.36E-07

rs4734879	A	G	White blood cell count	0.02203	0.004015	4.09E-08
rs4734879	A	G	Blood clot in the leg	0.002253	0.0003876	6.12E-09
rs4734879	A	G	Self-reported deep venous thrombosis	0.002319	0.0003833	1.46E-09
rs4734879	A	G	Coronary artery disease	0.029	0.0062	2.83E-06
rs7088799	G	T	Basophil percentage of granulocytes	0.01884	0.003555	1.16E-07
rs7088799	G	T	Granulocyte count	-0.03503	0.003643	6.87E-22
rs7088799	G	T	Granulocyte percentage of myeloid white cells	-0.02562	0.003625	1.58E-12
rs7088799	G	T	Lymphocyte percentage of white cells	0.01782	0.003614	8.23E-07
rs7088799	G	T	Mean platelet volume	-0.1322	0.003665	5.14E-285
rs7088799	G	T	Monocyte percentage of white cells	0.02105	0.003615	5.77E-09
rs7088799	G	T	Myeloid white cell count	-0.03397	0.003652	1.38E-20
rs7088799	G	T	Neutrophil count	-0.03616	0.003633	2.47E-23
rs7088799	G	T	Neutrophil percentage of white cells	-0.02388	0.00362	4.19E-11
rs7088799	G	T	Platelet count	0.0768	0.003698	8.75E-96
rs7088799	G	T	Platelet distribution width	-0.0547	0.003645	6.55E-51
rs7088799	G	T	Plateletcrit	0.0194	0.00371	1.71E-07
rs7088799	G	T	Sum basophil neutrophil counts	-0.0355	0.00364	1.77E-22
rs7088799	G	T	Sum neutrophil eosinophil counts	-0.03567	0.003637	1.04E-22
rs7088799	G	T	White blood cell count	-0.03342	0.003636	3.92E-20
rs7088799	G	T	Interleukin 10 levels	0.0852	0.01663	3.00E-07
rs7088799	G	T	Age at first live birth	0.01817	0.003996	5.41E-06
rs7088799	G	T	Age completed full time education	0.01488	0.002461	1.49E-09
rs7088799	G	T	Alcohol usually taken with meals	0.008371	0.001574	1.04E-07
rs7088799	G	T	Average weekly beer plus cider intake	-0.01214	0.002032	2.34E-09
rs7088799	G	T	Average weekly red wine intake	0.01187	0.002445	1.21E-06
rs7088799	G	T	Body mass index	-0.01392	0.002439	1.15E-08
rs7088799	G	T	Diastolic blood pressure	-0.01604	0.002498	1.34E-10
rs7088799	G	T	Height	0.009467	0.001752	6.51E-08
rs7088799	G	T	Impedance of arm left	0.01292	0.001741	1.18E-13
rs7088799	G	T	Impedance of arm right	0.01342	0.001733	9.94E-15
rs7088799	G	T	Impedance of whole body	0.01109	0.00188	3.67E-09
rs7088799	G	T	Leg fat percentage left	-0.007538	0.001541	1.01E-06
rs7088799	G	T	Leg fat percentage right	-0.007567	0.001561	1.26E-06
rs7088799	G	T	Mineral and other dietary supplements: zinc	0.002388	0.0004798	6.49E-07
rs7088799	G	T	Qualifications: A levels or as levels or equivalent	0.006394	0.001108	7.88E-09
rs7088799	G	T	Qualifications: college or university degree	0.005683	0.001156	8.80E-07
rs7088799	G	T	Qualifications: none	-0.007234	0.000937	1.16E-14
rs7088799	G	T	Self-reported hypertension	-0.005588	0.001082	2.39E-07

rs7088799	G	T	Sodium in urine	-0.01247	0.002401	2.03E-07
rs7088799	G	T	Usual walking pace	0.008633	0.001506	9.99E-09
rs7088799	G	T	Vascular or heart problems diagnosed by doctor: high bloo	-0.005631	0.001097	2.82E-07
rs7088799	G	T	Vascular or heart problems diagnosed by doctor: none of th	0.005425	0.001126	1.46E-06
rs7088799	G	T	Alkaline phosphatase	0.01	NA	8.08E-06
rs7088799	G	T	Years of educational attainment in males	0.022	0.004	5.79E-09
rs7088799	G	T	Years of educational attainment	0.014	0.003	9.40E-09
rs71361173	G	T	Interleukin 12p70 levels	0.111	0.02376	3.00E-06
rs72831623	A	G	Interferon gamma levels	-0.1803	0.0386	3.00E-06
rs72831623	A	G	Interleukin 12p70 levels	-0.1913	0.03679	2.00E-07
rs72831623	A	G	Interleukin 6 levels	-0.1973	0.03704	1.00E-07
rs782107	G	A	Interleukin 12p70 levels	0.075	0.01578	2.00E-06
rs79121401	C	T	Interleukin 12p70 levels	0.5548	0.1203	4.00E-06
rs117795020	A	G	Interleukin 13 levels	-0.3522	0.072	1.00E-06
rs117795020	A	G	Cause of death: other and unspecified cirrhosis of liver	0.01787	0.003647	9.79E-07
rs12623722	A	G	Interleukin 13 levels	-0.1185	0.0257	4.00E-06
rs139083458	C	T	Interleukin 13 levels	0.9902	0.212	3.00E-06
rs27949	T	C	Interleukin 13 levels	-0.1168	0.02501	3.00E-06
rs7073807	C	T	Interleukin 13 levels	0.1682	0.03539	2.00E-06
rs75995699	A	G	Interleukin 13 levels	-0.3319	0.07106	3.00E-06
rs9472168	A	G	Interleukin 13 levels	-0.4244	0.02479	1.00E-65
rs117217798	C	T	Interleukin 16 levels	0.2036	0.04415	4.00E-06
rs117916513	A	G	Interleukin 16 levels	-0.502	0.09903	4.00E-07
rs1255143	C	T	Interleukin 16 levels	0.1306	0.02422	7.00E-08
rs12765671	A	G	Interleukin 16 levels	-0.6023	0.1319	5.00E-06
rs144691581	A	G	Interleukin 16 levels	-0.4882	0.09631	4.00E-07
rs144691581	A	G	Cause of death: ill-defined sites within the digestive system	0.01236	0.002718	5.54E-06
rs1801020	G	A	Activated Partial Thromboplastin Time	NA	NA	1.24E-27
rs1801020	G	A	Activated partial thromboplastin time	NA	NA	1.26E-87
rs1801020	G	A	Activated partial thromboplastin time	NA	NA	1.55E-59
rs1801020	G	A	Factor XII antigen	NA	NA	4.81E-88
rs1801020	G	A	Interleukin 16 levels	0.1733	0.02787	5.00E-10
rs1801020	G	A	Arm fat-free mass left	-0.008426	0.001782	2.25E-06
rs1801020	G	A	Arm fat-free mass right	-0.00876	0.001746	5.25E-07
rs1801020	G	A	Arm predicted mass left	-0.008602	0.001775	1.26E-06
rs1801020	G	A	Arm predicted mass right	-0.008589	0.00174	7.95E-07
rs1801020	G	A	Comparative height size at age 10	-0.01204	0.001913	3.08E-10
rs1801020	G	A	Forced vital capacity	-0.01301	0.002275	1.09E-08

rs1801020	G	A	Forced vital capacity, best measure	-0.0143	0.002498	1.04E-08
rs1801020	G	A	Height	-0.01816	0.001991	7.35E-20
rs1801020	G	A	Trunk fat-free mass	-0.01027	0.001765	6.03E-09
rs1801020	G	A	Trunk predicted mass	-0.01031	0.001759	4.70E-09
rs1801020	G	A	Whole body fat-free mass	-0.008893	0.001772	5.21E-07
rs1801020	G	A	Whole body water mass	-0.008737	0.001775	8.52E-07
rs4253283	C	T	Interleukin 16 levels	0.146	0.02602	2.00E-08
rs4253283	C	T	Blood clot in the leg	0.002266	0.0003715	1.05E-09
rs4253283	C	T	Blood clot in the lung	0.001153	0.0002375	1.22E-06
rs4253283	C	T	Self-reported deep venous thrombosis	0.002234	0.0003674	1.19E-09
rs4253283	C	T	Self-reported pulmonary embolism + or - dvt	0.001229	0.0002378	2.38E-07
rs4513633	A	C	Interleukin 16 levels	-0.2239	0.04513	7.00E-07
rs4778636	A	G	Lymphocyte count	0.02915	0.006203	2.61E-06
rs4778636	A	G	Blood protein levels	NA	NA	4.00E-27
rs4778636	A	G	Interleukin 16 levels	-0.7272	0.0631	1.00E-30
rs4778636	A	G	Comparative height size at age 10	0.01492	0.002843	1.55E-07
rs4778636	A	G	Height	0.01538	0.002958	2.00E-07
rs9706053	C	T	Interleukin 16 levels	0.4582	0.09235	7.00E-07
rs117029961	A	G	Cause of death: pharynx, unspecified	0.01288	0.00252	3.24E-07
rs117556572	C	T	Interleukin 17 levels	0.5102	0.1092	3.00E-06
rs117556572	C	T	Cause of death: alcoholic cirrhosis of liver	-0.01432	0.002606	4.06E-08
rs1530455	C	T	Mean platelet volume	0.04211	0.003751	3.11E-29
rs1530455	C	T	Platelet count	-0.03895	0.003783	7.39E-25
rs1530455	C	T	Platelet distribution width	0.04323	0.003732	5.05E-31
rs1530455	C	T	Plateletcrit	-0.0227	0.003796	2.22E-09
rs1530455	C	T	Fibroblast growth factor basic levels	0.0777	0.0175	9.00E-06
rs1530455	C	T	Interleukin 10 levels	0.0793	0.01737	5.00E-06
rs1530455	C	T	Interleukin 17 levels	0.108	0.01737	5.00E-10
rs17106604	C	T	Interleukin 17 levels	0.1129	0.02262	6.00E-07
rs17282552	C	T	Interleukin 17 levels	0.2001	0.04054	8.00E-07
rs184080173	C	T	Interleukin 17 levels	0.2384	0.04703	4.00E-07
rs187475560	C	T	Interleukin 17 levels	0.2434	0.05211	3.00E-06
rs34120897	A	C	High light scatter percentage of red cells	-0.02665	0.005565	1.68E-06
rs34120897	A	C	High light scatter reticulocyte count	-0.02884	0.005566	2.19E-07
rs34120897	A	C	Reticulocyte count	-0.03101	0.005574	2.63E-08
rs34120897	A	C	Reticulocyte fraction of red cells	-0.02797	0.00557	5.12E-07
rs62191444	G	T	Interleukin 17 levels	0.1136	0.02463	4.00E-06
rs78612928	C	T	Interleukin 17 levels	0.1037	0.0222	3.00E-06

rs10414578	T	C	Interleukin 18 levels	-0.1771	0.03494	4.00E-07
rs116383510	A	C	Interleukin 18 levels	-0.5426	0.1059	3.00E-07
rs116383510	A	C	Treatment with anastrozole	-0.001464	0.0003278	7.97E-06
rs11700536	C	T	Interleukin 18 levels	0.1156	0.02507	4.00E-06
rs117266781	C	T	Interleukin 18 levels	0.6841	0.1465	3.00E-06
rs144841621	C	T	Interleukin 18 levels	0.518	0.1123	4.00E-06
rs144841621	C	T	Treatment with sulphasalazine	-0.002121	0.0004331	9.72E-07
rs17229943	A	C	Interleukin 18 levels	-0.312	0.04653	2.00E-11
rs1979967	C	T	Interleukin 18 levels	0.1402	0.02854	9.00E-07
rs2729385	G	A	Interleukin 18 levels	0.1231	0.02669	4.00E-06
rs385076	C	T	Interleukin 18 levels	0.2432	0.02496	2.00E-22
rs385076	C	T	Current tobacco smoking	-0.0063	0.001391	5.93E-06
rs4482818	A	G	Interleukin 18 levels	-0.1286	0.02414	1.00E-07
rs658805	G	A	Interleukin 18 levels	0.1226	0.02439	5.00E-07
rs71478720	C	T	Interleukin 18 levels	0.2669	0.0276	4.00E-22
rs71478720	C	T	Heel bone mineral density	-0.01907	0.003556	8.23E-08
rs78623212	C	T	Interleukin 18 levels	0.8705	0.1755	7.00E-07
rs78623212	C	T	Cause of death: appendix	-0.007275	0.001313	3.08E-08
rs78623212	C	T	Treatment with azithromycin	-0.0005717	0.0001284	8.55E-06
rs78716465	A	G	Interleukin 18 levels	-0.3265	0.06869	2.00E-06
rs115242021	A	C	Interleukin 1 beta levels	-0.2326	0.04628	5.00E-07
rs143319329	C	T	Interleukin 1 beta levels	0.2801	0.05893	2.00E-06
rs1942793	G	T	Interleukin 1 beta levels	0.0717	0.01571	5.00E-06
rs62015704	A	G	Interleukin 1 beta levels	-0.1082	0.02276	2.00E-06
rs9898641	C	T	Granulocyte count	-0.02151	0.003875	2.85E-08
rs9898641	C	T	Monocyte count	-0.03524	0.003857	6.35E-20
rs9898641	C	T	Myeloid white cell count	-0.02453	0.003885	2.72E-10
rs9898641	C	T	Neutrophil count	-0.02039	0.003866	1.33E-07
rs9898641	C	T	Platelet count	-0.01976	0.003938	5.25E-07
rs9898641	C	T	Platelet distribution width	0.03697	0.003882	1.66E-21
rs9898641	C	T	Sum basophil neutrophil counts	-0.02071	0.003872	8.83E-08
rs9898641	C	T	Sum eosinophil basophil counts	-0.01704	0.003855	9.83E-06
rs9898641	C	T	Sum neutrophil eosinophil counts	-0.02141	0.003869	3.14E-08
rs9898641	C	T	White blood cell count	-0.02624	0.00387	1.19E-11
rs9898641	C	T	Interleukin 1 beta levels	0.2032	0.04406	4.00E-06
rs11627423	A	C	Interleukin 1 receptor antagonist levels	-0.1171	0.02463	2.00E-06
rs12121840	C	T	Interleukin 1 receptor antagonist levels	0.2692	0.05663	2.00E-06
rs139005642	AA/	A	Interleukin 1 receptor antagonist levels	NA	NA	2.00E-06

rs2809154	C	T	Interleukin 1 receptor antagonist levels	0.1786	0.03873	4.00E-06
rs56134659	A	G	Interleukin 1 receptor antagonist levels	-0.1117	0.0235	2.00E-06
rs9623661	C	T	Interleukin 1 receptor antagonist levels	0.1966	0.04263	4.00E-06
rs10903540	A	G	Interleukin 2 levels	-0.1581	0.03428	4.00E-06
rs12051139	C	T	Interleukin 1 receptor antagonist levels	0.1074	0.02419	9.00E-06
rs12051139	C	T	Interleukin 2 levels	0.1131	0.02478	5.00E-06
rs13412535	A	G	Mean platelet volume	-0.02775	0.004338	1.59E-10
rs13412535	A	G	Fibroblast growth factor basic levels	-0.1112	0.02241	7.00E-07
rs13412535	A	G	Hepatocyte growth factor levels	-0.095	0.0214	9.00E-06
rs13412535	A	G	Interleukin 2 levels	-0.1764	0.03312	1.00E-07
rs13412535	A	G	Interleukin 6 levels	-0.1164	0.02159	7.00E-08
rs13412535	A	G	Mean platelet volume	0.02775	0.004362	2.00E-10
rs13412535	A	G	Platelet derived growth factor BB levels	-0.3352	0.02137	2.00E-55
rs13412535	A	G	Stem cell factor levels	-0.1067	0.02138	6.00E-07
rs1534019	C	T	Waist hip ratio adjusted for BMI	0.019	0.0043	7.20E-06
rs1534019	C	T	Total body bone mineral density	NA	NA	2.32E-11
rs1534019	C	T	Fracture of forearm	0.001277	0.0002415	1.26E-07
rs1534019	C	T	Fractured bone site: ankle	0.001372	0.0002897	2.19E-06
rs1534019	C	T	Fractured bone site: wrist	0.001925	0.0003439	2.17E-08
rs1534019	C	T	Fractured or broken bones in last 5 years	0.004654	0.0007263	1.48E-10
rs1534019	C	T	Heel bone mineral density	-0.08705	0.003137	3.57E-169
rs1534019	C	T	Heel bone mineral density left	-0.08786	0.004198	4.69E-97
rs1534019	C	T	Heel bone mineral density right	-0.08495	0.004172	5.15E-92
rs170117	C	T	Granulocyte count	-0.03371	0.005345	2.84E-10
rs170117	C	T	Hematocrit	0.04528	0.005259	7.33E-18
rs170117	C	T	Hemoglobin concentration	0.03576	0.005283	1.30E-11
rs170117	C	T	High light scatter percentage of red cells	-0.05328	0.005342	1.98E-23
rs170117	C	T	High light scatter reticulocyte count	-0.0365	0.005342	8.40E-12
rs170117	C	T	Immature fraction of reticulocytes	-0.05773	0.00529	9.96E-28
rs170117	C	T	Lymphocyte percentage of white cells	0.02771	0.005303	1.74E-07
rs170117	C	T	Mean corpuscular hemoglobin	-0.1166	0.00527	1.56E-108
rs170117	C	T	Mean corpuscular volume	-0.1248	0.005255	9.72E-125
rs170117	C	T	Myeloid white cell count	-0.03278	0.005357	9.39E-10
rs170117	C	T	Neutrophil count	-0.03435	0.005332	1.17E-10
rs170117	C	T	Neutrophil percentage of white cells	-0.02892	0.005312	5.19E-08
rs170117	C	T	Red blood cell count	0.1123	0.005296	7.09E-100
rs170117	C	T	Reticulocyte fraction of red cells	-0.03594	0.005348	1.81E-11
rs170117	C	T	Sum basophil neutrophil counts	-0.03438	0.00534	1.21E-10

rs170117	C	T	Sum neutrophil eosinophil counts	-0.03383	0.005337	2.32E-10
rs170117	C	T	White blood cell count	-0.02767	0.005337	2.15E-07
rs170117	C	T	Hematocrit Hct	NA	NA	7.24E-11
rs170117	C	T	Hemoglobin Hb	NA	NA	4.99E-09
rs170117	C	T	Mean corpuscular hemoglobin concentration	NA	NA	2.48E-18
rs170117	C	T	Mean corpuscular volume	NA	NA	4.31E-22
rs170117	C	T	Red blood cell count RBC	NA	NA	1.33E-31
rs2807544	A	G	Interleukin 2 levels	-0.1175	0.02516	3.00E-06
rs4634519	A	G	Interleukin 2 levels	-0.1261	0.027	3.00E-06
rs62124990	G	T	Interleukin 2 levels	0.6961	0.149	3.00E-06
rs62124990	G	T	Right eye myopia	-0.03087	0.006434	1.61E-06
rs7615304	A	G	Interleukin 2 levels	-0.1172	0.02396	1.00E-06
rs7615304	A	G	Heel bone mineral density	-0.02233	0.00324	5.58E-12
rs7615304	A	G	Heel bone mineral density left	-0.02579	0.004342	2.86E-09
rs7615304	A	G	Heel bone mineral density right	-0.02508	0.004313	6.04E-09
rs80336398	C	T	Interleukin 2 levels	0.4001	0.08566	3.00E-06
rs80336398	C	T	Treatment with otomize ear spray	0.001205	0.0002374	3.80E-07
rs115360066	A	G	Interleukin 2 receptor antagonist levels	-0.1867	0.03783	8.00E-07
rs117244812	A	G	Interleukin 2 receptor antagonist levels	-0.7064	0.1486	2.00E-06
rs12722497	C	A	Interleukin 2 receptor antagonist levels	0.6279	0.04844	2.00E-38
rs12722497	C	A	Strep throat	NA	NA	5.00E-07
rs185231391	C	T	Interleukin 2 receptor antagonist levels	0.8503	0.1738	1.00E-06
rs4733117	A	C	Interleukin 2 receptor antagonist levels	-0.1369	0.02931	3.00E-06
rs61705228	C	T	Interleukin 2 receptor antagonist levels	0.3303	0.07163	4.00E-06
rs61705228	C	T	Malignant neoplasm of rectum	-0.001121	0.0002429	3.95E-06
rs10512267	C	T	Interleukin 4 levels	0.0824	0.01608	3.00E-07
rs10512267	C	T	Sodium in urine	-0.01147	0.002522	5.40E-06
rs116705532	G	T	Interleukin 4 levels	0.4678	0.09841	2.00E-06
rs117146485	C	T	Interleukin 4 levels	0.2924	0.0626	3.00E-06
rs117146485	C	T	Interleukin 6 levels	0.2829	0.0625	6.00E-06
rs17713451	A	G	Interleukin 4 levels	-0.1274	0.02535	5.00E-07
rs6765768	A	G	Sitting height	0.008708	0.001944	7.47E-06
rs73023729	A	G	Interleukin 4 levels	-0.1796	0.03656	9.00E-07
rs7613691	A	G	Interleukin 4 levels	-0.1775	0.03849	4.00E-06
rs79597994	C	T	Interleukin 4 levels	0.5831	0.1264	4.00E-06
rs9508291	C	T	Interleukin 4 levels	0.1676	0.03588	3.00E-06
rs9941733	A	G	Interleukin 4 levels	-0.114	0.02298	7.00E-07
rs9941733	A	G	Platelet derived growth factor BB levels	-0.1161	0.02266	3.00E-07

rs11680908	A	G	Interleukin 5 levels	-0.2634	0.05541	2.00E-06
rs6737109	C	T	Interleukin 5 levels	0.116	0.0244	2.00E-06
rs72831687	A	G	Interleukin 5 levels	-0.5239	0.1102	2.00E-06
rs72831687	A	G	Cause of death: larynx, unspecified	0.02195	0.004516	1.19E-06
rs72831687	A	G	Cause of death: rheumatoid arthritis, unspecified	0.01566	0.003468	6.43E-06
rs73040130	C	T	Interleukin 5 levels	0.2638	0.05285	6.00E-07
rs7767396	A	G	Serum vascular endothelial growth factor VEGF	NA	NA	0
rs7767396	A	G	Interleukin 5 levels	-0.1515	0.02465	8.00E-10
rs114373846	C	T	Interleukin 6 levels	0.422	0.09035	3.00E-06
rs114373846	C	T	Cause of death: ischaemic cardiomyopathy	-0.01117	0.00234	1.86E-06
rs1333040	C	T	Coronary artery disease	-0.1549	0.0172	2.44E-19
rs1333040	C	T	Coronary artery disease	-0.1878	0.03243	6.99E-09
rs1333040	C	T	Coronary artery disease	-0.1575	0.01454	2.73E-27
rs1333040	C	T	Coronary artery disease	-0.1414	0.01969	6.94E-13
rs1333040	C	T	Myocardial infarction	-0.1246	0.02047	1.17E-09
rs1333040	C	T	Abdominal aortic aneurysm	NA	NA	1.60E-07
rs1333040	C	T	Coronary artery calcification CAC	NA	NA	1.67E-12
rs1333040	C	T	Coronary artery calcification CAC	NA	NA	3.59E-06
rs1333040	C	T	Coronary artery calcification CAC	NA	NA	1.64E-09
rs1333040	C	T	Coronary artery calcification CAC excluding previous myo	NA	NA	8.47E-12
rs1333040	C	T	Coronary artery disease	NA	NA	1.00E-07
rs1333040	C	T	Coronary artery disease	NA	NA	2.73E-27
rs1333040	C	T	Glaucoma primary open angle glaucoma	NA	NA	4.88E-09
rs1333040	C	T	Intracranial aneurysm	NA	NA	1.40E-10
rs1333040	C	T	Intracranial aneurysm	NA	NA	1.50E-22
rs1333040	C	T	Lung cancer squamous cell carcinoma	NA	NA	2.28E-08
rs1333040	C	T	Myocardial infarction	NA	NA	4.10E-15
rs1333040	C	T	Coronary artery disease	-0.1274	0.0111	1.23E-30
rs1333040	C	T	Coronary artery disease	-0.1304	0.0098	0
rs1333040	C	T	Interleukin 6 levels	0.0738	0.0158	3.00E-06
rs1333040	C	T	Intracranial aneurysm	-0.2546	0.03938	1.00E-10
rs1333040	C	T	Intracranial aneurysm	-0.2776	0.0285	2.00E-22
rs1333040	C	T	Squamous cell lung carcinoma	0.09363	0.01887	7.00E-07
rs1333040	C	T	Acute myocardial infarction	-0.00176	0.0002642	2.69E-11
rs1333040	C	T	Angina pectoris	-0.001698	0.0002934	7.15E-09
rs1333040	C	T	Chronic ischaemic heart disease	-0.004564	0.0003905	1.49E-31
rs1333040	C	T	Fathers age at death	0.01646	0.002852	7.81E-09
rs1333040	C	T	Haemorrhoids	0.001716	0.0003801	6.34E-06

rs1333040	C	T	Illnesses of father: heart disease	-0.01224	0.00123	2.54E-23
rs1333040	C	T	Illnesses of father: none of the above, group 1	0.00799	0.001278	3.99E-10
rs1333040	C	T	Illnesses of mother: heart disease	-0.006512	0.001014	1.38E-10
rs1333040	C	T	Illnesses of siblings: heart disease	-0.004538	0.0008245	3.72E-08
rs1333040	C	T	Medication for cholesterol, blood pressure or diabetes: cho	-0.00913	0.001538	2.92E-09
rs1333040	C	T	Medication for pain relief, constipation, heartburn: aspirin	-0.005548	0.0008559	9.09E-11
rs1333040	C	T	Self-reported angina	-0.004108	0.0004298	1.20E-21
rs1333040	C	T	Self-reported heart attack or myocardial infarction	-0.003163	0.0003677	7.80E-18
rs1333040	C	T	Treatment with aspirin	-0.005304	0.0008332	1.94E-10
rs1333040	C	T	Treatment with atorvastatin	-0.002416	0.0004216	1.01E-08
rs1333040	C	T	Treatment with bisoprolol	-0.001356	0.0002977	5.28E-06
rs1333040	C	T	Treatment with clopidogrel	-0.001048	0.000196	8.97E-08
rs1333040	C	T	Treatment with nicorandil	-0.0007423	0.0001399	1.13E-07
rs1333040	C	T	Treatment with simvastatin	-0.003961	0.0007807	3.90E-07
rs1333040	C	T	Vascular or heart problems diagnosed by doctor: angina	-0.004135	0.0004305	7.64E-22
rs1333040	C	T	Vascular or heart problems diagnosed by doctor: heart attac	-0.003156	0.0003695	1.33E-17
rs1333040	C	T	Coronary artery disease	-0.1242	0.01243	1.78E-23
rs1333040	C	T	Intracranial aneurysm	NA	NA	1.00E-10
rs1333040	C	T	Intracranial aneurysm	NA	NA	2.00E-22
rs1333040	C	T	Coronary artery disease	-0.1347	0.007044	1.60E-81
rs1333040	C	T	Coronary artery disease	-0.1332	0.0058	8.52E-118
rs13412535	A	G	Mean platelet volume	-0.02775	0.004338	1.59E-10
rs13412535	A	G	Fibroblast growth factor basic levels	-0.1112	0.02241	7.00E-07
rs13412535	A	G	Hepatocyte growth factor levels	-0.095	0.0214	9.00E-06
rs13412535	A	G	Interleukin 2 levels	-0.1764	0.03312	1.00E-07
rs13412535	A	G	Interleukin 6 levels	-0.1164	0.02159	7.00E-08
rs13412535	A	G	Mean platelet volume	0.02775	0.004362	2.00E-10
rs13412535	A	G	Platelet derived growth factor BB levels	-0.3352	0.02137	2.00E-55
rs13412535	A	G	Stem cell factor levels	-0.1067	0.02138	6.00E-07
rs72831623	A	G	Interferon gamma levels	-0.1803	0.0386	3.00E-06
rs72831623	A	G	Interleukin 12p70 levels	-0.1913	0.03679	2.00E-07
rs72831623	A	G	Interleukin 6 levels	-0.1973	0.03704	1.00E-07
rs73273528	C	T	Interleukin 6 levels	0.2672	0.05462	1.00E-06
rs73273528	C	T	Cause of death: organ-limited amyloidosis	-0.009213	0.001366	1.62E-11
rs76856708	C	T	Interleukin 6 levels	0.3289	0.07042	3.00E-06
rs117509142	C	T	Interleukin 7 levels	0.327	0.06879	2.00E-06
rs117509142	C	T	Cause of death: vascular dementia, unspecified	0.007698	0.00165	3.14E-06
rs141425475	C	T	Interleukin 7 levels	0.4781	0.1024	3.00E-06

rs144701438	A	G	Interleukin 7 levels	-0.4819	0.09852	1.00E-06
rs17091524	C	T	Interleukin 7 levels	0.4924	0.1036	2.00E-06
rs218260	C	T	Granulocyte count	-0.02954	0.004336	9.64E-12
rs218260	C	T	Hematocrit	0.04209	0.004266	5.84E-23
rs218260	C	T	Hemoglobin concentration	0.03345	0.004285	5.87E-15
rs218260	C	T	High light scatter percentage of red cells	-0.04643	0.004333	8.55E-27
rs218260	C	T	High light scatter reticulocyte count	-0.03093	0.004333	9.46E-13
rs218260	C	T	Immature fraction of reticulocytes	-0.0492	0.004291	1.95E-30
rs218260	C	T	Lymphocyte percentage of white cells	0.02181	0.004302	4.00E-07
rs218260	C	T	Mean corpuscular hemoglobin	-0.1074	0.004275	3.08E-139
rs218260	C	T	Mean corpuscular hemoglobin concentration	-0.01987	0.004184	2.04E-06
rs218260	C	T	Mean corpuscular volume	-0.1151	0.004263	1.20E-160
rs218260	C	T	Myeloid white cell count	-0.02939	0.004347	1.37E-11
rs218260	C	T	Neutrophil count	-0.03035	0.004325	2.28E-12
rs218260	C	T	Neutrophil percentage of granulocytes	-0.01917	0.004323	9.19E-06
rs218260	C	T	Neutrophil percentage of white cells	-0.02474	0.004309	9.43E-09
rs218260	C	T	Red blood cell count	0.1027	0.004296	2.79E-126
rs218260	C	T	Reticulocyte fraction of red cells	-0.03269	0.004338	4.83E-14
rs218260	C	T	Sum basophil neutrophil counts	-0.03041	0.004332	2.24E-12
rs218260	C	T	Sum neutrophil eosinophil counts	-0.02954	0.004329	8.92E-12
rs218260	C	T	White blood cell count	-0.02473	0.004329	1.12E-08
rs28793375	C	T	Interleukin 5 levels	0.1657	0.0363	5.00E-06
rs28793375	C	T	Interleukin 7 levels	0.1638	0.03552	4.00E-06
rs4320361	G	T	Interleukin 7 levels	0.3245	0.02488	7.00E-39
rs62006410	C	T	Interleukin 7 levels	0.1557	0.03039	3.00E-07
rs75904417	A	C	Interleukin 13 levels	-0.1515	0.03371	7.00E-06
rs75904417	A	C	Interleukin 7 levels	-0.1698	0.03471	1.00E-06
rs77318030	C	T	Granulocyte colony stimulating factor levels	0.2045	0.04302	2.00E-06
rs77981494	C	T	Interleukin 7 levels	0.5178	0.1059	1.00E-06
rs77981494	C	T	Cause of death: emphysema, unspecified	0.009693	0.002072	2.94E-06
rs78346957	A	G	Interleukin 13 levels	-0.4491	0.09994	7.00E-06
rs78346957	A	G	Interleukin 7 levels	-0.4588	0.1005	5.00E-06
rs11634944	C	T	Interleukin 8 levels	0.1214	0.02482	1.00E-06
rs12075	A	G	Basophil count	0.03469	0.003533	9.14E-23
rs12075	A	G	Basophil percentage of granulocytes	0.02609	0.003537	1.62E-13
rs12075	A	G	Basophil percentage of white cells	0.02827	0.003506	7.43E-16
rs12075	A	G	Granulocyte count	0.01866	0.003624	2.64E-07
rs12075	A	G	Monocyte count	0.03344	0.003605	1.77E-20

rs12075	A	G	Monocyte percentage of white cells	0.0202	0.003596	1.93E-08
rs12075	A	G	Myeloid white cell count	0.022	0.003634	1.42E-09
rs12075	A	G	Neutrophil count	0.01796	0.003615	6.78E-07
rs12075	A	G	Sum basophil neutrophil counts	0.01807	0.003621	6.01E-07
rs12075	A	G	Sum eosinophil basophil counts	0.01945	0.003604	6.81E-08
rs12075	A	G	Sum neutrophil eosinophil counts	0.0186	0.003618	2.73E-07
rs12075	A	G	White blood cell count	0.0203	0.003618	2.01E-08
rs12075	A	G	Duffy system Fya antigen	NA	NA	1.30E-29
rs12075	A	G	Fasting serum MCP 1 pgmL in children	NA	NA	1.31E-21
rs12075	A	G	Monocyte chemoattractant protein 1	NA	NA	7.43E-102
rs12075	A	G	Monocyte chemoattractant protein 1 in obese children	NA	NA	1.30E-21
rs12075	A	G	Monocyte count	NA	NA	1.14E-09
rs12075	A	G	White blood cell count	NA	NA	4.92E-24
rs12075	A	G	Basophil percentage of granulocytes	0.02609	0.00355	2.00E-13
rs12075	A	G	Basophil percentage of white cells	0.02827	0.003503	7.00E-16
rs12075	A	G	Educational attainment	0.022	0.004497	1.00E-06
rs12075	A	G	Eotaxin levels	-0.1671	0.01561	1.00E-26
rs12075	A	G	Growth regulated protein alpha levels	-0.3751	0.02385	1.00E-55
rs12075	A	G	Inflammatory biomarkers	NA	NA	4.00E-51
rs12075	A	G	Interleukin 8 levels	-0.12	0.02367	4.00E-07
rs12075	A	G	Monocyte chemoattractant protein 1	NA	NA	1.00E-21
rs12075	A	G	Monocyte chemoattractant protein 1 levels	-0.2185	0.01557	1.00E-44
rs12075	A	G	Monocyte count	0.03344	0.00361	2.00E-20
rs12075	A	G	Myeloid white cell count	0.022	0.0036	1.00E-09
rs12075	A	G	Obesity related traits	0.1	0.01044	1.00E-21
rs12075	A	G	White blood cell count	-1.27	0.1256	5.00E-24
rs12075	A	G	White blood cell count basophil	0.03469	0.003532	9.00E-23
rs12075	A	G	Chemokine ccl2	NA	NA	4.00E-51
rs12075	A	G	Leukocyte count	NA	NA	5.00E-24
rs141926526	A	C	Interleukin 8 levels	-0.6149	0.1316	3.00E-06
rs2673604	C	A	Alzheimers disease APOE3 homozygotes	NA	NA	4.07E-06
rs2673604	C	A	Interleukin 8 levels	0.1266	0.02552	7.00E-07
rs41294750	C	T	Interleukin 9 levels	0.3514	0.07393	2.00E-06
rs4880409	C	T	Interleukin 9 levels	0.3355	0.07183	3.00E-06
rs61867538	C	T	Interleukin 9 levels	0.3566	0.07733	4.00E-06
rs61867538	C	T	Cause of death: emphysema, unspecified	-0.007382	0.001424	2.23E-07
rs7232268	A	G	Mean platelet volume	0.09738	0.009796	2.75E-23
rs7232268	A	G	Platelet distribution width	0.0638	0.009733	5.58E-11

rs7232268	A	G	Interleukin 9 levels	-0.2759	0.05907	3.00E-06
rs7232268	A	G	Macrophage inflammatory protein 1a levels	-0.2821	0.0604	3.00E-06
rs7232268	A	G	Open wound of head	0.001633	0.0003567	4.68E-06
rs7242404	A	G	Interleukin 9 levels	-0.1228	0.02629	3.00E-06
rs7242404	A	G	Macrophage inflammatory protein 1a levels	-0.1211	0.02675	6.00E-06
rs76963786	C	T	Interleukin 9 levels	0.2865	0.05652	4.00E-07
rs10809307	C	T	Interferon gamma induced protein 10 levels	0.1305	0.0283	4.00E-06
rs113831257	A	G	Interferon gamma induced protein 10 levels	-0.3592	0.06482	3.00E-08
rs11626201	A	C	Interferon gamma induced protein 10 levels	-0.1162	0.02445	2.00E-06
rs11626201	A	C	Height	0.008999	0.001768	3.60E-07
rs11626201	A	C	Relative age of first facial hair	0.007337	0.001622	6.12E-06
rs34383175	C	T	Interferon gamma induced protein 10 levels	0.3153	0.06633	2.00E-06
rs397816	T	C	Interferon gamma induced protein 10 levels	-0.1237	0.02506	8.00E-07
rs75970138	A	G	Interferon gamma induced protein 10 levels	-0.485	0.102	2.00E-06
rs75970138	A	G	Self-reported parathyroid hyperplasia or adenoma	0.001029	0.0002211	3.27E-06
rs7645625	G	T	Interferon gamma induced protein 10 levels	0.1086	0.02355	4.00E-06
rs79848609	A	C	Interferon gamma induced protein 10 levels	-0.2603	0.05299	9.00E-07
rs8112909	A	G	Interferon gamma induced protein 10 levels	-0.1426	0.03	2.00E-06
rs9450351	C	T	Interferon gamma induced protein 10 levels	0.2768	0.0483	1.00E-08
rs10835056	G	T	Macrophage inflammatory protein 1a levels	0.1194	0.02556	3.00E-06
rs116615337	A	G	Macrophage inflammatory protein 1a levels	-0.1278	0.028	5.00E-06
rs12690897	A	G	Macrophage inflammatory protein 1a levels	-0.1248	0.02625	2.00E-06
rs34771762	A	G	Macrophage inflammatory protein 1a levels	-0.249	0.05238	2.00E-06
rs57786342	A	G	Macrophage inflammatory protein 1a levels	-0.1314	0.02849	4.00E-06
rs57786342	A	G	Vascular or heart problems diagnosed by doctor: high bloo	0.006459	0.001346	1.60E-06
rs57786342	A	G	Vascular or heart problems diagnosed by doctor: none of th	-0.006726	0.001382	1.14E-06
rs60198979	A	G	Macrophage inflammatory protein 1a levels	-0.2146	0.04594	3.00E-06
rs6900267	A	C	Eosinophil count	0.04087	0.00846	1.36E-06
rs6900267	A	C	Eosinophil percentage of white cells	0.03822	0.008455	6.17E-06
rs6900267	A	C	Sum eosinophil basophil counts	0.0385	0.008468	5.46E-06
rs6900267	A	C	Macrophage inflammatory protein 1a levels	-0.2429	0.052	3.00E-06
rs7232268	A	G	Mean platelet volume	0.09738	0.009796	2.75E-23
rs7232268	A	G	Platelet distribution width	0.0638	0.009733	5.58E-11
rs7232268	A	G	Interleukin 9 levels	-0.2759	0.05907	3.00E-06
rs7232268	A	G	Macrophage inflammatory protein 1a levels	-0.2821	0.0604	3.00E-06
rs7232268	A	G	Open wound of head	0.001633	0.0003567	4.68E-06
rs113010081	C	T	Lymphocyte count	0.02951	0.005589	1.29E-07
rs113010081	C	T	Lymphocyte percentage of white cells	0.03013	0.00553	5.08E-08

rs113010081	C	T	Ulcerative colitis	0.1578	0.0345	4.89E-06
rs113010081	C	T	Inflammatory bowel disease	0.09223	0.0168	4.00E-08
rs113010081	C	T	Macrophage inflammatory protein 1b levels	0.5954	0.02363	4.00E-140
rs113010081	C	T	Ulcerative colitis	0.1271	0.02074	9.00E-10
rs113010081	C	T	Ulcerative colitis	NA	NA	2.00E-06
rs113010081	C	T	Rheumatoid arthritis	-0.131	0.02688	3.00E-06
rs113010081	C	T	Rheumatoid arthritis	-0.131	0.02688	3.00E-06
rs113010081	C	T	Coronary artery disease	0.05225	0.0113	3.80E-06
rs113010081	C	T	Coronary artery disease	0.0459	0.0097	2.08E-06
rs113877493	C	T	Macrophage inflammatory protein 1b levels	0.6124	0.02181	2.00E-173
rs116237296	A	G	Macrophage inflammatory protein 1b levels	-0.5437	0.1096	7.00E-07
rs116237296	A	G	Treatment with loratadine product	0.0008439	0.0001785	2.28E-06
rs17138331	A	G	Macrophage inflammatory protein 1b levels	-0.1391	0.02926	2.00E-06
rs281749	T	C	Macrophage inflammatory protein 1b levels	-0.0799	0.01711	3.00E-06
rs72791296	C	T	Macrophage inflammatory protein 1b levels	0.2369	0.04674	4.00E-07
rs72799710	C	T	Macrophage inflammatory protein 1b levels	0.1014	0.02171	3.00E-06
rs74810984	C	T	Macrophage inflammatory protein 1b levels	0.2206	0.04641	2.00E-06
rs76582507	A	G	Macrophage inflammatory protein 1b levels	-0.3175	0.06798	3.00E-06
rs76583883	G	T	Macrophage inflammatory protein 1b levels	0.2317	0.05076	5.00E-06
rs76776296	A	G	Macrophage inflammatory protein 1b levels	-0.2997	0.06005	6.00E-07
rs9793308	A	G	Macrophage inflammatory protein 1b levels	-0.0835	0.01788	3.00E-06
rs11087905	A	C	Eotaxin levels	-0.0941	0.01872	5.00E-07
rs112347425	C	T	Monocyte percentage of white cells	-0.02659	0.005945	7.73E-06
rs112347425	C	T	Eotaxin levels	0.158	0.02749	9.00E-09
rs12075	A	G	Basophil count	0.03469	0.003533	9.14E-23
rs12075	A	G	Basophil percentage of granulocytes	0.02609	0.003537	1.62E-13
rs12075	A	G	Basophil percentage of white cells	0.02827	0.003506	7.43E-16
rs12075	A	G	Granulocyte count	0.01866	0.003624	2.64E-07
rs12075	A	G	Monocyte count	0.03344	0.003605	1.77E-20
rs12075	A	G	Monocyte percentage of white cells	0.0202	0.003596	1.93E-08
rs12075	A	G	Myeloid white cell count	0.022	0.003634	1.42E-09
rs12075	A	G	Neutrophil count	0.01796	0.003615	6.78E-07
rs12075	A	G	Sum basophil neutrophil counts	0.01807	0.003621	6.01E-07
rs12075	A	G	Sum eosinophil basophil counts	0.01945	0.003604	6.81E-08
rs12075	A	G	Sum neutrophil eosinophil counts	0.0186	0.003618	2.73E-07
rs12075	A	G	White blood cell count	0.0203	0.003618	2.01E-08
rs12075	A	G	Duffy system Fya antigen	NA	NA	1.30E-29
rs12075	A	G	Fasting serum MCP 1 pgmL in children	NA	NA	1.31E-21

rs12075	A	G	Monocyte chemoattractant protein 1	NA	NA	7.43E-102
rs12075	A	G	Monocyte chemoattractant protein 1 in obese children	NA	NA	1.30E-21
rs12075	A	G	Monocyte count	NA	NA	1.14E-09
rs12075	A	G	White blood cell count	NA	NA	4.92E-24
rs12075	A	G	Basophil percentage of granulocytes	0.02609	0.00355	2.00E-13
rs12075	A	G	Basophil percentage of white cells	0.02827	0.003503	7.00E-16
rs12075	A	G	Educational attainment	0.022	0.004497	1.00E-06
rs12075	A	G	Eotaxin levels	-0.1671	0.01561	1.00E-26
rs12075	A	G	Growth regulated protein alpha levels	-0.3751	0.02385	1.00E-55
rs12075	A	G	Inflammatory biomarkers	NA	NA	4.00E-51
rs12075	A	G	Interleukin 8 levels	-0.12	0.02367	4.00E-07
rs12075	A	G	Monocyte chemoattractant protein 1	NA	NA	1.00E-21
rs12075	A	G	Monocyte chemoattractant protein 1 levels	-0.2185	0.01557	1.00E-44
rs12075	A	G	Monocyte count	0.03344	0.00361	2.00E-20
rs12075	A	G	Myeloid white cell count	0.022	0.0036	1.00E-09
rs12075	A	G	Obesity related traits	0.1	0.01044	1.00E-21
rs12075	A	G	White blood cell count	-1.27	0.1256	5.00E-24
rs12075	A	G	White blood cell count basophil	0.03469	0.003532	9.00E-23
rs12075	A	G	Chemokine ccl2	NA	NA	4.00E-51
rs12075	A	G	Leukocyte count	NA	NA	5.00E-24
rs1476670	A	C	Eotaxin levels	-0.1007	0.02184	4.00E-06
rs2024050	G	A	Basophil count	-0.02625	0.00573	4.62E-06
rs2024050	G	A	Basophil percentage of granulocytes	-0.02814	0.005734	9.20E-07
rs2024050	G	A	Basophil percentage of white cells	-0.03044	0.005686	8.59E-08
rs2024050	G	A	Eosinophil count	-0.06782	0.005836	3.26E-31
rs2024050	G	A	Eosinophil percentage of granulocytes	-0.06893	0.005854	5.28E-32
rs2024050	G	A	Eosinophil percentage of white cells	-0.07228	0.00583	2.67E-35
rs2024050	G	A	Neutrophil percentage of granulocytes	0.06683	0.005855	3.53E-30
rs2024050	G	A	Sum eosinophil basophil counts	-0.06581	0.005843	2.02E-29
rs2024050	G	A	Eotaxin levels	0.1728	0.03015	1.00E-08
rs2210755	C	T	Eotaxin levels	0.1104	0.02419	5.00E-06
rs2211994	C	T	Eotaxin levels	0.0885	0.01773	6.00E-07
rs2228467	C	T	Eosinophil count	0.06971	0.007408	4.94E-21
rs2228467	C	T	Eosinophil percentage of granulocytes	0.05538	0.007434	9.30E-14
rs2228467	C	T	Eosinophil percentage of white cells	0.0552	0.007403	8.82E-14
rs2228467	C	T	Granulocyte count	0.03429	0.007464	4.35E-06
rs2228467	C	T	Granulocyte percentage of myeloid white cells	-0.1271	0.00742	9.96E-66
rs2228467	C	T	Monocyte count	0.1734	0.007416	6.99E-121

rs2228467	C	T	Monocyte percentage of white cells	0.1553	0.007401	1.06E-97
rs2228467	C	T	Myeloid white cell count	0.05385	0.007481	6.09E-13
rs2228467	C	T	Neutrophil percentage of granulocytes	-0.04929	0.007435	3.38E-11
rs2228467	C	T	Sum eosinophil basophil counts	0.06697	0.007416	1.70E-19
rs2228467	C	T	Sum neutrophil eosinophil counts	0.034	0.007455	5.10E-06
rs2228467	C	T	White blood cell count	0.04845	0.007453	7.99E-11
rs2228467	C	T	Fasting serum eotaxin pgmL in children	NA	NA	1.89E-06
rs2228467	C	T	Monocyte count	NA	NA	2.39E-08
rs2228467	C	T	Blood protein levels	0.5514	0.08349	4.00E-11
rs2228467	C	T	Cerebrospinal fluid levels of Alzheimers disease related pr	NA	NA	4.00E-18
rs2228467	C	T	Eosinophil counts	0.06971	0.007409	5.00E-21
rs2228467	C	T	Eosinophil percentage of granulocytes	0.05538	0.007429	9.00E-14
rs2228467	C	T	Eosinophil percentage of white cells	0.0552	0.007405	9.00E-14
rs2228467	C	T	Eotaxin levels	0.4163	0.0291	2.00E-46
rs2228467	C	T	Granulocyte percentage of myeloid white cells	0.1271	0.00742	1.00E-65
rs2228467	C	T	Monocyte chemoattractant protein 1 levels	0.2637	0.02898	9.00E-20
rs2228467	C	T	Monocyte count	0.3	0.0577	2.00E-07
rs2228467	C	T	Monocyte count	0.1734	0.007416	7.00E-121
rs2228467	C	T	Monocyte percentage of white cells	0.1553	0.0074	1.00E-97
rs2228467	C	T	Myeloid white cell count	0.05385	0.007478	6.00E-13
rs2228467	C	T	Neutrophil percentage of granulocytes	0.04929	0.007416	3.00E-11
rs2228467	C	T	Sum eosinophil basophil counts	0.06697	0.00743	2.00E-19
rs2419841	C	T	Eotaxin levels	0.1277	0.02798	5.00E-06
rs5746492	A	G	Eotaxin levels	-0.0954	0.02069	4.00E-06
rs5754733	A	C	Bipolar disorder	0.1182	0.0262	2.89E-06
rs5754733	A	C	Eotaxin levels	-0.1042	0.0213	1.00E-06
rs59808887	C	T	Eotaxin levels	0.1673	0.03582	3.00E-06
rs75426604	A	C	Eotaxin levels	-0.1366	0.02925	3.00E-06
rs79722574	C	T	Eotaxin levels	0.1113	0.02275	1.00E-06
rs80341932	A	G	Eotaxin levels	-0.1016	0.02048	7.00E-07
rs9317045	A	C	Eotaxin levels	-0.1182	0.02368	6.00E-07
rs9317045	A	C	Monocyte chemoattractant protein 1 levels	-0.1134	0.02386	2.00E-06
rs10145849	A	G	Monocyte chemoattractant protein 1 levels	-0.0755	0.01616	3.00E-06
rs10744620	C	T	Monocyte chemoattractant protein 1 levels	0.0788	0.01611	1.00E-06
rs111995966	G	T	Monocyte chemoattractant protein 1 levels	0.1452	0.03109	3.00E-06
rs12073356	A	G	High light scatter percentage of red cells	0.03606	0.007402	1.11E-06
rs12073356	A	G	High light scatter reticulocyte count	0.03488	0.007401	2.45E-06
rs12073356	A	G	Reticulocyte count	0.03551	0.007412	1.66E-06

rs12073356	A	G	Reticulocyte fraction of red cells	0.0382	0.007408	2.52E-07
rs12075	A	G	Basophil count	0.03469	0.003533	9.14E-23
rs12075	A	G	Basophil percentage of granulocytes	0.02609	0.003537	1.62E-13
rs12075	A	G	Basophil percentage of white cells	0.02827	0.003506	7.43E-16
rs12075	A	G	Granulocyte count	0.01866	0.003624	2.64E-07
rs12075	A	G	Monocyte count	0.03344	0.003605	1.77E-20
rs12075	A	G	Monocyte percentage of white cells	0.0202	0.003596	1.93E-08
rs12075	A	G	Myeloid white cell count	0.022	0.003634	1.42E-09
rs12075	A	G	Neutrophil count	0.01796	0.003615	6.78E-07
rs12075	A	G	Sum basophil neutrophil counts	0.01807	0.003621	6.01E-07
rs12075	A	G	Sum eosinophil basophil counts	0.01945	0.003604	6.81E-08
rs12075	A	G	Sum neutrophil eosinophil counts	0.0186	0.003618	2.73E-07
rs12075	A	G	White blood cell count	0.0203	0.003618	2.01E-08
rs12075	A	G	Duffy system Fya antigen	NA	NA	1.30E-29
rs12075	A	G	Fasting serum MCP 1 pgmL in children	NA	NA	1.31E-21
rs12075	A	G	Monocyte chemoattractant protein 1	NA	NA	7.43E-102
rs12075	A	G	Monocyte chemoattractant protein 1 in obese children	NA	NA	1.30E-21
rs12075	A	G	Monocyte count	NA	NA	1.14E-09
rs12075	A	G	White blood cell count	NA	NA	4.92E-24
rs12075	A	G	Basophil percentage of granulocytes	0.02609	0.00355	2.00E-13
rs12075	A	G	Basophil percentage of white cells	0.02827	0.003503	7.00E-16
rs12075	A	G	Educational attainment	0.022	0.004497	1.00E-06
rs12075	A	G	Eotaxin levels	-0.1671	0.01561	1.00E-26
rs12075	A	G	Growth regulated protein alpha levels	-0.3751	0.02385	1.00E-55
rs12075	A	G	Inflammatory biomarkers	NA	NA	4.00E-51
rs12075	A	G	Interleukin 8 levels	-0.12	0.02367	4.00E-07
rs12075	A	G	Monocyte chemoattractant protein 1	NA	NA	1.00E-21
rs12075	A	G	Monocyte chemoattractant protein 1 levels	-0.2185	0.01557	1.00E-44
rs12075	A	G	Monocyte count	0.03344	0.00361	2.00E-20
rs12075	A	G	Myeloid white cell count	0.022	0.0036	1.00E-09
rs12075	A	G	Obesity related traits	0.1	0.01044	1.00E-21
rs12075	A	G	White blood cell count	-1.27	0.1256	5.00E-24
rs12075	A	G	White blood cell count basophil	0.03469	0.003532	9.00E-23
rs12075	A	G	Chemokine ccl2	NA	NA	4.00E-51
rs12075	A	G	Leukocyte count	NA	NA	5.00E-24
rs146522229	C	T	Monocyte chemoattractant protein 1 levels	0.5976	0.1179	4.00E-07
rs146522229	C	T	Treatment with calcium carbonate+colecalciferol 1.25g or [illegible]	-0.001122	0.0002326	1.39E-06
rs2036297	A	G	Basophil count	0.02236	0.003676	1.19E-09

rs2036297	A	G	Basophil percentage of granulocytes	0.01715	0.00368	3.15E-06
rs2036297	A	G	Basophil percentage of white cells	0.02071	0.003648	1.36E-08
rs2036297	A	G	Granulocyte percentage of myeloid white cells	0.0253	0.003753	1.57E-11
rs2036297	A	G	Monocyte count	-0.01713	0.00375	4.90E-06
rs2036297	A	G	Monocyte percentage of white cells	-0.02486	0.003741	3.03E-11
rs2036297	A	G	Mouth or teeth dental problems: mouth ulcers	-0.006241	0.0007775	9.98E-16
rs2036297	A	G	Mouth or teeth dental problems: none of the above	0.005855	0.001252	2.93E-06
rs2288370	C	T	Granulocyte percentage of myeloid white cells	-0.026	0.003707	2.30E-12
rs2288370	C	T	Monocyte count	0.03996	0.003704	3.92E-27
rs2288370	C	T	Monocyte percentage of white cells	0.0338	0.003696	5.90E-20
rs2712431	A	C	Eosinophil count	-0.03712	0.003828	3.11E-22
rs2712431	A	C	Eosinophil percentage of granulocytes	-0.04635	0.00384	1.51E-33
rs2712431	A	C	Eosinophil percentage of white cells	-0.04568	0.003823	6.64E-33
rs2712431	A	C	Granulocyte count	0.02254	0.003855	5.00E-09
rs2712431	A	C	Granulocyte percentage of myeloid white cells	-0.05088	0.003832	3.22E-40
rs2712431	A	C	Lymphocyte percentage of white cells	-0.03139	0.003823	2.24E-16
rs2712431	A	C	Mean platelet volume	0.01986	0.003879	3.05E-07
rs2712431	A	C	Monocyte count	0.07791	0.00383	5.47E-92
rs2712431	A	C	Monocyte percentage of white cells	0.06845	0.003822	9.51E-72
rs2712431	A	C	Myeloid white cell count	0.03066	0.003864	2.12E-15
rs2712431	A	C	Neutrophil count	0.02646	0.003845	5.92E-12
rs2712431	A	C	Neutrophil percentage of granulocytes	0.04473	0.00384	2.40E-31
rs2712431	A	C	Neutrophil percentage of white cells	0.01905	0.00383	6.55E-07
rs2712431	A	C	Sum basophil neutrophil counts	0.02615	0.003852	1.13E-11
rs2712431	A	C	Sum eosinophil basophil counts	-0.03422	0.003832	4.23E-19
rs2712431	A	C	Sum neutrophil eosinophil counts	0.02303	0.003849	2.18E-09
rs2712431	A	C	White blood cell count	0.02413	0.003849	3.64E-10
rs2712431	A	C	Monocyte chemoattractant protein 1 levels	-0.0787	0.01724	5.00E-06
rs56212190	C	T	Monocyte chemoattractant protein 1 levels	0.181	0.037	1.00E-06
rs7197349	A	G	Monocyte chemoattractant protein 1 levels	-0.0968	0.02072	3.00E-06
rs7632755	A	G	Granulocyte percentage of myeloid white cells	0.0417	0.00703	3.00E-09
rs7632755	A	G	Monocyte count	-0.04498	0.007023	1.50E-10
rs7632755	A	G	Monocyte percentage of white cells	-0.0496	0.007008	1.47E-12
rs7632755	A	G	Mouth or teeth dental problems: mouth ulcers	0.007925	0.001464	6.20E-08
rs9317045	A	C	Eotaxin levels	-0.1182	0.02368	6.00E-07
rs9317045	A	C	Monocyte chemoattractant protein 1 levels	-0.1134	0.02386	2.00E-06
rs10892381	C	T	Monocyte chemoattractant protein 3 levels	0.2412	0.04758	4.00E-07
rs62492260	G	T	Granulocyte count	0.03275	0.005389	1.22E-09

rs62492260	G	T	Myeloid white cell count	0.0322	0.005401	2.50E-09
rs62492260	G	T	Neutrophil count	0.03359	0.005375	4.14E-10
rs62492260	G	T	Red cell distribution width	-0.03268	0.005308	7.44E-10
rs62492260	G	T	Sum basophil neutrophil counts	0.03387	0.005384	3.18E-10
rs62492260	G	T	Sum neutrophil eosinophil counts	0.03268	0.00538	1.24E-09
rs62492260	G	T	White blood cell count	0.03074	0.005379	1.09E-08
rs62492260	G	T	Monocyte chemoattractant protein 3 levels	0.2788	0.05865	2.00E-06
rs62492260	G	T	Heel bone mineral density	-0.02294	0.004646	7.91E-07
rs73669117	A	G	Cause of death: asthma, unspecified	-0.01183	0.002278	2.14E-07
rs73669117	A	G	Cause of death: other specified respiratory disorders	-0.008272	0.001518	5.22E-08
rs111607343	A	G	Monokine induced by gamma interferon levels	-0.521	0.1115	3.00E-06
rs11177248	A	G	Monokine induced by gamma interferon levels	-0.3073	0.06664	4.00E-06
rs112337562	G	T	Monokine induced by gamma interferon levels	0.37	0.07922	3.00E-06
rs112861654	A	G	Monokine induced by gamma interferon levels	-0.2765	0.05318	2.00E-07
rs117831247	C	T	Monokine induced by gamma interferon levels	0.8334	0.1753	2.00E-06
rs139010077	C	T	Monokine induced by gamma interferon levels	0.4322	0.09372	4.00E-06
rs139010077	C	T	Cause of death: ischaemic cardiomyopathy	-0.01246	0.002452	3.87E-07
rs41272086	G	A	Monokine induced by gamma interferon levels	0.2226	0.04129	7.00E-08
rs41272086	G	A	Coronary artery disease	0.06915	0.01517	5.27E-06
rs41272086	G	A	Coronary artery disease	0.06927	0.01159	2.30E-09
rs41272086	G	A	Coronary artery disease	0.0684	0.0098	3.70E-12
rs55876513	G	T	Monokine induced by gamma interferon levels	0.166	0.02554	8.00E-11
rs5752128	C	T	Monokine induced by gamma interferon levels	0.1685	0.03654	4.00E-06
rs62562991	A	G	Monokine induced by gamma interferon levels	-0.6236	0.1264	8.00E-07
rs6679677	A	C	Basophil percentage of granulocytes	0.0306	0.005773	1.16E-07
rs6679677	A	C	Basophil percentage of white cells	0.03124	0.005722	4.77E-08
rs6679677	A	C	Granulocyte count	-0.04239	0.005918	7.87E-13
rs6679677	A	C	Lymphocyte count	-0.05556	0.005932	7.57E-21
rs6679677	A	C	Myeloid white cell count	-0.04242	0.005932	8.59E-13
rs6679677	A	C	Neutrophil count	-0.04258	0.005901	5.35E-13
rs6679677	A	C	Sum basophil neutrophil counts	-0.04266	0.005911	5.31E-13
rs6679677	A	C	Sum neutrophil eosinophil counts	-0.04246	0.005909	6.68E-13
rs6679677	A	C	White blood cell count	-0.05549	0.005907	5.72E-21
rs6679677	A	C	Amoxicillin clavulanate drug induced liver injury	NA	NA	8.70E-06
rs6679677	A	C	Crohns disease	NA	NA	4.95E-09
rs6679677	A	C	Crohns disease	NA	NA	2.03E-15
rs6679677	A	C	Drug induced liver injury all cholestatic DILI cases	NA	NA	1.35E-06
rs6679677	A	C	Hypothyroidism	NA	NA	2.80E-13

rs6679677	A	C	Juvenile idiopathic arthritis	NA	NA	1.07E-06
rs6679677	A	C	Juvenile idiopathic arthritis including oligoarticular and rhe	NA	NA	3.19E-25
rs6679677	A	C	Oligoarticular juvenile idiopathic arthritis	NA	NA	1.11E-18
rs6679677	A	C	Rheumatoid arthritis	NA	NA	5.70E-42
rs6679677	A	C	Rheumatoid arthritis	NA	NA	4.39E-70
rs6679677	A	C	Rheumatoid factor negative polyarticular juvenile idiopathi	NA	NA	3.45E-11
rs6679677	A	C	Selective immunoglobulin A deficiency IgAD	NA	NA	6.00E-42
rs6679677	A	C	Type 1 diabetes	NA	NA	1.40E-41
rs6679677	A	C	Type 1 diabetes	NA	NA	1.27E-40
rs6679677	A	C	Crohns disease	NA	NA	5.00E-09
rs6679677	A	C	Crohns disease	-0.2172	0.0429	4.17E-07
rs6679677	A	C	Antineutrophil cytoplasmic antibody associated vasculitis	NA	NA	2.00E-08
rs6679677	A	C	Crohns disease	NA	NA	2.00E-15
rs6679677	A	C	Hypothyroidism	0.3082	0.04225	3.00E-13
rs6679677	A	C	Monokine induced by gamma interferon levels	-0.162	0.03298	9.00E-07
rs6679677	A	C	Pediatric autoimmune diseases	NA	NA	8.00E-11
rs6679677	A	C	Rheumatoid arthritis	0.6831	0.06622	6.00E-25
rs6679677	A	C	Rheumatoid arthritis	NA	NA	6.00E-42
rs6679677	A	C	Systemic lupus erythematosus	0.3293	0.04103	1.00E-15
rs6679677	A	C	Type 1 diabetes	0.6366	0.06326	8.00E-24
rs6679677	A	C	Type 1 diabetes	0.5988	0.05675	5.00E-26
rs6679677	A	C	Type 1 diabetes	NA	NA	1.00E-40
rs6679677	A	C	Diabetes diagnosed by doctor	0.004567	0.000857	9.88E-08
rs6679677	A	C	Insulin-dependent diabetes mellitus	0.0008135	0.0001433	1.37E-08
rs6679677	A	C	Long-standing illness, disability or infirmity	0.01442	0.001896	2.88E-14
rs6679677	A	C	Medication for cholesterol, blood pressure or diabetes: insu	0.005448	0.000686	2.00E-15
rs6679677	A	C	Number of self-reported non-cancer illnesses	0.01682	0.003302	3.49E-07
rs6679677	A	C	Number of treatments or medications taken	0.01842	0.003206	9.17E-09
rs6679677	A	C	Other rheumatoid arthritis	0.001127	0.0002141	1.40E-07
rs6679677	A	C	Other serious medical condition or disability diagnosed by	0.01176	0.001634	6.12E-13
rs6679677	A	C	Self-reported diabetes	0.003986	0.0007771	2.90E-07
rs6679677	A	C	Self-reported hyperthyroidism or thyrotoxicosis	0.002618	0.0003469	4.42E-14
rs6679677	A	C	Self-reported hypothyroidism or myxoedema	0.0201	0.000853	1.07E-122
rs6679677	A	C	Self-reported pernicious anaemia	0.001065	0.0002202	1.31E-06
rs6679677	A	C	Self-reported rheumatoid arthritis	0.002726	0.0004193	7.94E-11
rs6679677	A	C	Self-reported type 1 diabetes	0.0005363	0.0001168	4.38E-06
rs6679677	A	C	Started insulin within one year diagnosis of diabetes	0.05595	0.005856	1.43E-21
rs6679677	A	C	Taking other prescription medications	0.01598	0.002002	1.44E-15

rs6679677	A	C	Treatment with folic acid product	0.001789	0.0003626	8.02E-07
rs6679677	A	C	Treatment with insulin	0.003193	0.0004787	2.57E-11
rs6679677	A	C	Treatment with insulin product	0.00387	0.0003958	1.42E-22
rs6679677	A	C	Treatment with levothyroxine sodium	0.01513	0.0007863	1.91E-82
rs6679677	A	C	Treatment with methotrexate	0.002405	0.0002905	1.25E-16
rs6679677	A	C	Treatment with thyroxine product	0.005396	0.0004285	2.40E-36
rs6679677	A	C	Treatment with thyroxine sodium	0.001188	0.0002112	1.85E-08
rs6679677	A	C	Rheumatoid arthritis	0.5933	0.02256	3.10E-149
rs6679677	A	C	Rheumatoid arthritis	0.5933	0.02256	2.10E-149
rs6679677	A	C	Rheumatoid arthritis	0.6627	0.03811	4.39E-70
rs6679677	A	C	Arthritis rheumatoid	NA	NA	6.00E-25
rs6679677	A	C	Arthritis rheumatoid	NA	NA	6.00E-42
rs6679677	A	C	Diabetes mellitus type 1	NA	NA	8.00E-24
rs6679677	A	C	Diabetes mellitus type 1	NA	NA	5.00E-26
rs6679677	A	C	Diabetes mellitus type 1	NA	NA	1.00E-40
rs6679677	A	C	Hypothyroidism	NA	NA	3.00E-13
rs6679677	A	C	Coronary artery disease	0.0437	0.0088	7.07E-07
rs77086208	C	T	Monokine induced by gamma interferon levels	0.3226	0.06996	4.00E-06
rs816960	T	C	Monokine induced by gamma interferon levels	-0.1224	0.02435	5.00E-07
rs116303454	A	G	CTACK levels	-0.383	0.082	3.00E-06
rs145902143	A	G	CTACK levels	-0.2838	0.05802	1.00E-06
rs2070074	A	G	CTACK levels	-0.4467	0.03768	2.00E-32
rs3766110	A	C	Neuroticism	NA	NA	7.38E-06
rs3766110	A	C	CTACK levels	-0.1287	0.02791	4.00E-06
rs3766110	A	C	Blood clot in the leg	0.002224	0.0004088	5.36E-08
rs3766110	A	C	Phlebitis and thrombophlebitis	0.001065	0.0001989	8.67E-08
rs3766110	A	C	Self-reported deep venous thrombosis	0.002261	0.0004044	2.24E-08
rs55764737	C	T	CTACK levels	0.5313	0.09746	5.00E-08
rs57338032	A	G	CTACK levels	-0.1583	0.03172	6.00E-07
rs7333764	C	T	CTACK levels	0.2773	0.05937	3.00E-06
rs76395525	A	G	CTACK levels	-0.5277	0.1079	1.00E-06
rs76395525	A	G	Doctor diagnosed chronic bronchitis	0.01037	0.002263	4.62E-06
rs112072646	A	G	RANTES levels	-0.4286	0.08587	6.00E-07
rs147509526	C	T	RANTES levels	0.358	0.07216	7.00E-07
rs4940620	A	G	RANTES levels	-0.2494	0.05408	4.00E-06
rs62438851	A	G	RANTES levels	-0.1957	0.04117	2.00E-06
rs7000423	C	T	RANTES levels	0.1318	0.02535	2.00E-07
rs72793342	A	G	Eosinophil count	-0.0228	0.004459	3.18E-07

rs72793342	A	G	Sum eosinophil basophil counts	-0.02129	0.004464	1.86E-06
rs72793342	A	G	RANTES levels	-0.1487	0.0304	1.00E-06
rs72793342	A	G	Diastolic blood pressure	0.01699	0.00309	3.80E-08
rs74472919	C	T	RANTES levels	0.3313	0.06034	4.00E-08
rs74472919	C	T	Cause of death: chronic myeloid leukaemia	-0.00725	0.001518	1.82E-06
rs75613039	C	T	RANTES levels	0.37	0.08106	5.00E-06
rs75613039	C	T	Atrial fibrillation and flutter	0.003789	0.0007469	3.92E-07
rs818452	T	C	RANTES levels	-0.2381	0.05009	2.00E-06
rs818452	T	C	Cause of death: perforation of intestine	0.005774	0.001153	5.61E-07
rs1113500	G	T	Growth regulated protein alpha levels	0.1174	0.0247	2.00E-06
rs118158560	A	G	Growth regulated protein alpha levels	-0.2703	0.05787	3.00E-06
rs118158560	A	G	Fibroblastic disorders	0.003392	0.0003857	1.43E-18
rs118158560	A	G	Self-reported dupuytrens contracture	0.0008339	0.0001395	2.28E-09
rs12075	A	G	Basophil count	0.03469	0.003533	9.14E-23
rs12075	A	G	Basophil percentage of granulocytes	0.02609	0.003537	1.62E-13
rs12075	A	G	Basophil percentage of white cells	0.02827	0.003506	7.43E-16
rs12075	A	G	Granulocyte count	0.01866	0.003624	2.64E-07
rs12075	A	G	Monocyte count	0.03344	0.003605	1.77E-20
rs12075	A	G	Monocyte percentage of white cells	0.0202	0.003596	1.93E-08
rs12075	A	G	Myeloid white cell count	0.022	0.003634	1.42E-09
rs12075	A	G	Neutrophil count	0.01796	0.003615	6.78E-07
rs12075	A	G	Sum basophil neutrophil counts	0.01807	0.003621	6.01E-07
rs12075	A	G	Sum eosinophil basophil counts	0.01945	0.003604	6.81E-08
rs12075	A	G	Sum neutrophil eosinophil counts	0.0186	0.003618	2.73E-07
rs12075	A	G	White blood cell count	0.0203	0.003618	2.01E-08
rs12075	A	G	Duffy system Fya antigen	NA	NA	1.30E-29
rs12075	A	G	Fasting serum MCP 1 pgmL in children	NA	NA	1.31E-21
rs12075	A	G	Monocyte chemoattractant protein 1	NA	NA	7.43E-102
rs12075	A	G	Monocyte chemoattractant protein 1 in obese children	NA	NA	1.30E-21
rs12075	A	G	Monocyte count	NA	NA	1.14E-09
rs12075	A	G	White blood cell count	NA	NA	4.92E-24
rs12075	A	G	Basophil percentage of granulocytes	0.02609	0.00355	2.00E-13
rs12075	A	G	Basophil percentage of white cells	0.02827	0.003503	7.00E-16
rs12075	A	G	Educational attainment	0.022	0.004497	1.00E-06
rs12075	A	G	Eotaxin levels	-0.1671	0.01561	1.00E-26
rs12075	A	G	Growth regulated protein alpha levels	-0.3751	0.02385	1.00E-55
rs12075	A	G	Inflammatory biomarkers	NA	NA	4.00E-51
rs12075	A	G	Interleukin 8 levels	-0.12	0.02367	4.00E-07

rs12075	A	G	Monocyte chemoattractant protein 1	NA	NA	1.00E-21
rs12075	A	G	Monocyte chemoattractant protein 1 levels	-0.2185	0.01557	1.00E-44
rs12075	A	G	Monocyte count	0.03344	0.00361	2.00E-20
rs12075	A	G	Myeloid white cell count	0.022	0.0036	1.00E-09
rs12075	A	G	Obesity related traits	0.1	0.01044	1.00E-21
rs12075	A	G	White blood cell count	-1.27	0.1256	5.00E-24
rs12075	A	G	White blood cell count basophil	0.03469	0.003532	9.00E-23
rs12075	A	G	Chemokine ccl2	NA	NA	4.00E-51
rs12075	A	G	Leukocyte count	NA	NA	5.00E-24
rs140734053	A	G	Growth regulated protein alpha levels	-0.7257	0.1574	4.00E-06
rs185768063	A	G	Growth regulated protein alpha levels	-0.3998	0.07506	1.00E-07
rs185768063	A	G	Treatment with migril tablet	-0.0009278	0.0002071	7.44E-06
rs188345231	C	T	Mean corpuscular hemoglobin concentration	-0.05912	0.01263	2.85E-06
rs188345231	C	T	Growth regulated protein alpha levels	0.623	0.1351	4.00E-06
rs508977	T	G	Granulocyte count	-0.0193	0.004246	5.49E-06
rs508977	T	G	Myeloid white cell count	-0.0189	0.004257	9.03E-06
rs508977	T	G	Neutrophil count	-0.01908	0.004235	6.63E-06
rs508977	T	G	Sum basophil neutrophil counts	-0.01918	0.004242	6.11E-06
rs508977	T	G	Sum neutrophil eosinophil counts	-0.01942	0.00424	4.66E-06
rs508977	T	G	Growth regulated protein alpha levels	-0.3802	0.02806	8.00E-42
rs62024303	A	G	3 hydroxypropylmercapturic acid levels in smokers	NA	NA	3.00E-07
rs62024303	A	G	Growth regulated protein alpha levels	-0.3053	0.06621	4.00E-06
rs78653452	G	T	Growth regulated protein alpha levels	0.7362	0.1505	1.00E-06
rs78653452	G	T	Neoplasm of uncertain or unknown behaviour of endocrine	-0.0008282	0.0001692	9.85E-07
rs10474392	A	G	Stromal cell derived factor 1 alpha levels	-0.0962	0.01967	1.00E-06
rs12407262	A	G	Stromal cell derived factor 1 alpha levels	-0.1179	0.02557	4.00E-06
rs13400104	A	G	Stromal cell derived factor 1 alpha levels	-0.0647	0.01417	5.00E-06
rs13400104	A	G	Mean time to correctly identify matches	0.01681	0.00334	4.84E-07
rs139840550	A	G	Stromal cell derived factor 1 alpha levels	-0.1834	0.03977	4.00E-06
rs149893336	A	G	Stromal cell derived factor 1 alpha levels	-0.5034	0.1103	5.00E-06
rs4581824	G	T	Stromal cell derived factor 1 alpha levels	0.0701	0.01501	3.00E-06
rs482700	A	G	Stromal cell derived factor 1 alpha levels	-0.0893	0.01879	2.00E-06
rs67689854	A	C	Mean platelet volume	0.02553	0.005386	2.13E-06
rs67689854	A	C	Stromal cell derived factor 1 alpha levels	-0.0681	0.01458	3.00E-06
rs67689854	A	C	Arm fat mass left	-0.02061	0.003559	7.05E-09
rs67689854	A	C	Arm fat mass right	-0.02086	0.003558	4.57E-09
rs67689854	A	C	Arm fat percentage left	-0.01519	0.002749	3.27E-08
rs67689854	A	C	Arm fat percentage right	-0.01627	0.002762	3.88E-09

rs67689854	A	C	Basal metabolic rate	-0.01159	0.002408	1.49E-06
rs67689854	A	C	Body fat percentage	-0.0172	0.002784	6.53E-10
rs67689854	A	C	Height	-0.01767	0.002581	7.64E-12
rs67689854	A	C	Hip circumference	-0.01899	0.003597	1.29E-07
rs67689854	A	C	Leg fat mass left	-0.01594	0.00287	2.81E-08
rs67689854	A	C	Leg fat mass right	-0.01667	0.002903	9.35E-09
rs67689854	A	C	Leg fat percentage right	-0.01064	0.0023	3.70E-06
rs67689854	A	C	Leg fat-free mass left	-0.01204	0.002391	4.76E-07
rs67689854	A	C	Leg predicted mass left	-0.0117	0.002375	8.47E-07
rs67689854	A	C	Malignant melanoma of skin	0.0009788	0.0001983	7.96E-07
rs67689854	A	C	Number of self-reported cancers	0.00561	0.001134	7.48E-07
rs67689854	A	C	Other malignant neoplasms of skin	0.00255	0.0004328	3.83E-09
rs67689854	A	C	Relative age of first facial hair	0.01061	0.002367	7.36E-06
rs67689854	A	C	Self-reported basal cell carcinoma	0.001825	0.000351	1.98E-07
rs67689854	A	C	Self-reported malignant melanoma	0.002795	0.0003235	5.54E-18
rs67689854	A	C	Trunk fat mass	-0.02659	0.003656	3.56E-13
rs67689854	A	C	Trunk fat percentage	-0.02314	0.003338	4.10E-12
rs67689854	A	C	Waist circumference	-0.01694	0.003217	1.39E-07
rs67689854	A	C	Weight	-0.01878	0.003173	3.29E-09
rs67689854	A	C	Whole body fat mass	-0.02308	0.003543	7.33E-11
rs10834997	A	G	Tumor necrosis factor alpha levels	-0.1247	0.02549	1.00E-06
rs111332265	A	G	Tumor necrosis factor alpha levels	-0.3766	0.07591	7.00E-07
rs115669577	A	G	Tumor necrosis factor alpha levels	-0.9889	0.2004	8.00E-07
rs79105320	A	G	Tumor necrosis factor alpha levels	-0.5605	0.1215	4.00E-06
rs8121916	A	C	Tumor necrosis factor alpha levels	-0.1306	0.02796	3.00E-06
rs8121916	A	C	Arm fat-free mass left	0.008279	0.001789	3.69E-06
rs8121916	A	C	Arm fat-free mass right	0.008005	0.001753	4.96E-06
rs8121916	A	C	Arm predicted mass right	0.007725	0.001747	9.77E-06
rs8121916	A	C	Basal metabolic rate	0.008729	0.001866	2.88E-06
rs8121916	A	C	Leg fat-free mass left	0.00835	0.001853	6.57E-06
rs8121916	A	C	Leg fat-free mass right	0.008716	0.001852	2.52E-06
rs8121916	A	C	Leg predicted mass left	0.008302	0.00184	6.42E-06
rs8121916	A	C	Leg predicted mass right	0.008504	0.00184	3.80E-06
rs8121916	A	C	Trunk fat mass	0.01253	0.002833	9.66E-06
rs8121916	A	C	Weight	0.01185	0.002459	1.43E-06
rs10925040	C	T	Tumor necrosis factor beta levels	0.1755	0.03757	3.00E-06
rs753274	T	C	Tumor necrosis factor beta levels	-0.1736	0.03717	3.00E-06
rs7629875	A	G	Tumor necrosis factor beta levels	-0.3766	0.07699	1.00E-06

rs11618126	A	G	TRAIL levels	-0.8908	0.1821	1.00E-06
rs11618126	A	G	Injury of muscle and tendon at forearm level	-0.0007628	0.0001713	8.43E-06
rs11618126	A	G	Treatment with migril tablet	-0.0007012	0.0001554	6.45E-06
rs11657269	A	G	TRAIL levels	-0.1188	0.02603	5.00E-06
rs11699445	G	T	TRAIL levels	0.0746	0.01597	3.00E-06
rs13185784	A	G	TRAIL levels	-0.0846	0.01835	4.00E-06
rs13278062	G	T	Age-related macular degeneration	NA	NA	4.50E-11
rs13278062	G	T	Age-related macular degeneration	NA	NA	7.22E-10
rs13278062	G	T	Neovascularization	NA	NA	3.38E-12
rs13278062	G	T	Advanced age related macular degeneration	NA	NA	3.00E-15
rs13278062	G	T	Advanced age related macular degeneration choroidal neov	NA	NA	3.38E-12
rs13278062	G	T	Exudative age related macular degeneration	NA	NA	1.03E-12
rs13278062	G	T	Advanced age related macular degeneration	NA	NA	5.00E-11
rs13278062	G	T	Age related macular degeneration	-0.3148	0.04415	1.00E-12
rs13278062	G	T	Age related macular degeneration	-0.1398	0.01771	3.00E-15
rs13278062	G	T	TRAIL levels	0.0801	0.0158	4.00E-07
rs13278062	G	T	Macular degeneration	NA	NA	1.00E-12
rs141603697	C	T	Treatment with bromelains	-0.001175	0.0002312	3.69E-07
rs146783010	A	G	TRAIL levels	-0.6016	0.1318	5.00E-06
rs148051545	C	T	TRAIL levels	0.3921	0.08503	4.00E-06
rs193112415	C	T	TRAIL levels	1.042	0.0625	2.00E-62
rs193112415	C	T	Treatment with senokot 7.5mg tablet	0.001028	0.0001895	5.73E-08
rs57396456	C	T	TRAIL levels	0.5626	0.05155	1.00E-27
rs62093514	C	T	TRAIL levels	1.062	0.0554	7.00E-82
rs747324	C	T	TRAIL levels	0.0855	0.01799	2.00E-06
rs74778900	C	T	TRAIL levels	0.5906	0.05358	3.00E-28
rs75928541	A	G	TRAIL levels	-0.275	0.05964	4.00E-06
rs75928541	A	G	Cause of death: malignant neoplasm of thyroid gland	0.006318	0.00134	2.47E-06
rs75928541	A	G	Treatment with st johns wort or hypericum	0.001642	0.0003672	7.71E-06
rs79287178	A	G	Granulocyte percentage of myeloid white cells	0.04931	0.01056	3.04E-06
rs79287178	A	G	Monocyte percentage of white cells	-0.05306	0.01053	4.63E-07
rs79287178	A	G	Platelet count	0.06418	0.01076	2.44E-09
rs79287178	A	G	Plateletcrit	0.06704	0.0108	5.49E-10
rs79287178	A	G	Platelet count	0.06418	0.0107	2.00E-09
rs79287178	A	G	Plateletcrit	0.06704	0.01078	5.00E-10
rs79287178	A	G	TRAIL levels	0.4317	0.04201	9.00E-25
rs79287178	A	G	Cause of death: oropharynx, unspecified	0.008031	0.001769	5.73E-06
rs79287178	A	G	Treatment with rosuvastatin	0.002902	0.0005783	5.22E-07

rs113218956	A	G	Disorders of vestibular function	0.002179	0.0004847	6.96E-06
rs118055855	C	T	Macrophage Migration Inhibitory Factor levels	0.6907	0.1498	4.00E-06
rs12594190	A	G	Macrophage Migration Inhibitory Factor levels	-0.1355	0.02673	4.00E-07
rs13142904	C	T	Macrophage Migration Inhibitory Factor levels	0.223	0.04353	3.00E-07
rs141009259	C	T	Macrophage Migration Inhibitory Factor levels	0.6178	0.13	2.00E-06
rs5751777	C	T	Injury of eye and orbit	0.0002007	4.43E-05	5.87E-06
rs78098071	C	T	Macrophage Migration Inhibitory Factor levels	0.4867	0.09361	2.00E-07
rs78098071	C	T	Self-reported pericardial problem	0.00118	0.0002618	6.55E-06
rs112783231	A	G	Interferon gamma levels	-0.2408	0.05066	2.00E-06
rs113600793	A	C	Neutrophil percentage of granulocytes	-0.04009	0.008899	6.63E-06
rs113600793	A	C	Sum eosinophil basophil counts	0.03978	0.008865	7.22E-06
rs113600793	A	C	Interferon gamma levels	-0.1829	0.03723	9.00E-07
rs113600793	A	C	Interleukin 12p70 levels	-0.1814	0.03634	6.00E-07
rs113600793	A	C	Interleukin 6 levels	-0.1771	0.03588	8.00E-07
rs11843756	G	T	Interferon gamma levels	0.184	0.03939	3.00E-06
rs11843756	G	T	Cause of death: bronchiectasis	0.01527	0.002882	1.20E-07
rs12420286	C	T	Interferon gamma levels	0.2376	0.04999	2.00E-06
