# Supplementary material for: Inflammatory Cytokines and Risk of Ischemic Stroke: A Mendelian Randomization Study
Source: Front Pharmacol. 2022 Jan 17;12:779899. doi: 10.3389/fphar.2021.779899 (PMC8801801; doi:10.3389/fphar.2021.779899)
Supplement: Supplementary file 4 [file DataSheet1.PDF]

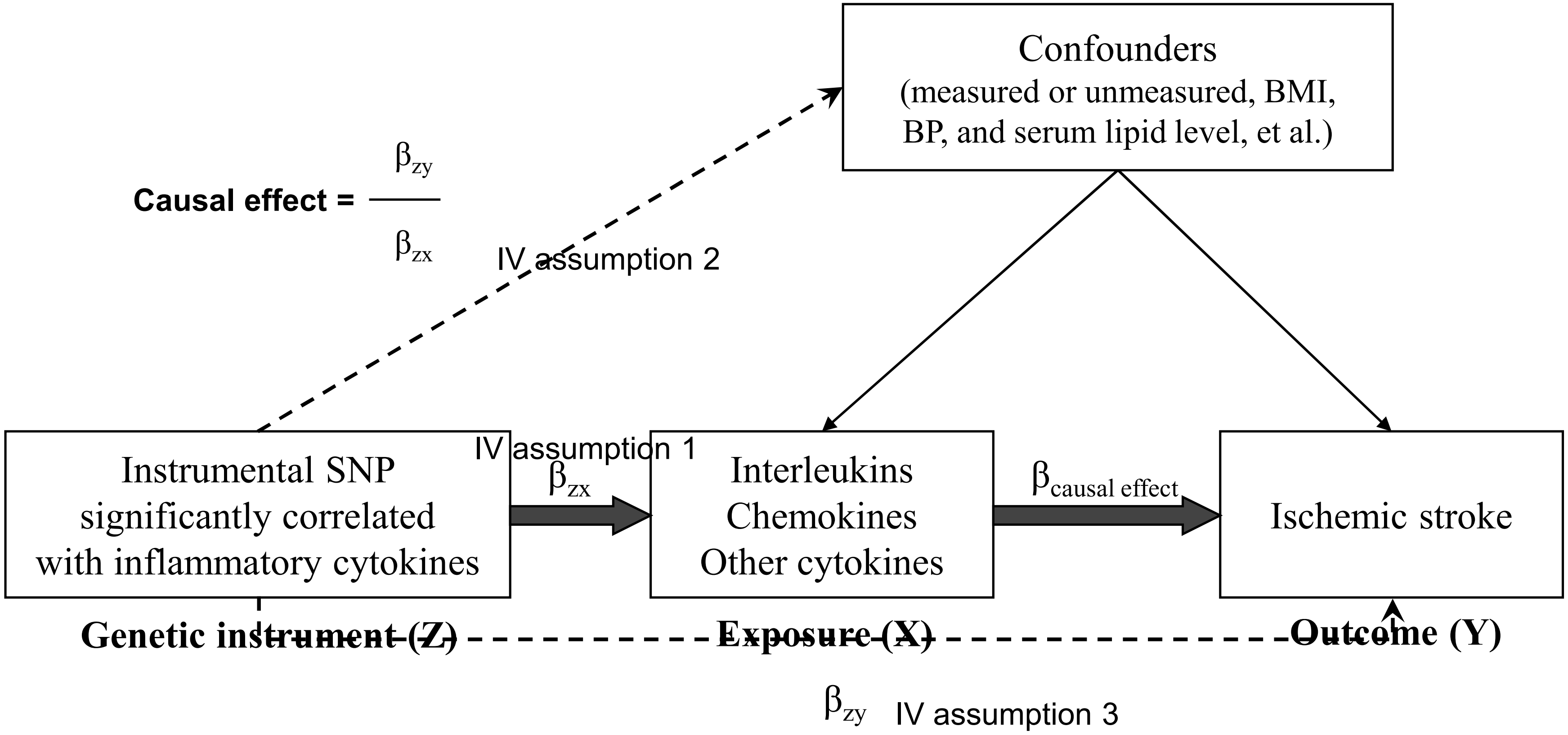

Figure S1. Instrumental variable (IV) assumptions of Mendelian randomization.

SNP indicates single nucleotide polymorphism; BMI, body mass index; BP, blood pressure.
